# All-atom normal mode dynamics of HIV-1 capsid

**DOI:** 10.1371/journal.pcbi.1006456

**Published:** 2018-09-18

**Authors:** Hyuntae Na, Guang Song

**Affiliations:** 1 Department of Computer Science, Penn State Harrisburg, Middletown, Pennsylvania, United States of America; 2 Department of Computer Science, Iowa State University, Ames, Iowa, United States of America; 3 Program of Bioinformatics and Computational Biology, Iowa State University, Ames, Iowa, United States of America; University at Buffalo, The State University of New York, UNITED STATES

## Abstract

Dynamics of biomolecular assemblies offer invaluable insights into their functional mechanisms. For extremely large biomolecular systems, such as HIV-1 capsid that has nearly 5 millions atoms, obtaining its normal mode dynamics using even coarse-grained models can be a challenging task. In this work, we have successfully carried out a normal mode analysis of an entire HIV-1 capsid in full all-atom details. This is made possible through our newly developed BOSE (Block of Selected Elasticity) model that is founded on the principle of resonance discovered in our recent work. The resonance principle makes it possible to most efficiently compute the vibrations of a whole capsid at any given frequency by projecting the motions of component capsomeres into a narrow subspace. We have conducted also assessments of the quality of the BOSE modes by comparing them with benchmark modes obtained directly from the original Hessian matrix. Our all-atom normal mode dynamics study of the HIV-1 capsid reveals the dynamic role of the pentamers in stabilizing the capsid structure and is in agreement with experimental findings that suggest capsid disassembly and uncoating start when the pentamers become destabilized. Our results on the dynamics of hexamer pores suggest that nucleotide transport should take place mostly at hexamers near pentamers, especially at the larger hemispherical end.

## Author summary

Supramolecular assemblies are large biomolecular complexes composed of hundreds or even thousands of protein chains. They function as molecular machines or as large containers that store or facilitate the chemical reactions of other molecules. Whatever they do, their functional mechanisms are tightly linked to their structures and intrinsic dynamics. Recently, due to breakthroughs in experimental techniques, many supramolecular assemblies have been determined, such as the capsid of human immunodeficiency virus (HIV) that is composed of nearly 5 millions of atoms. Computational studies of these systems are challenging due to their extremely large sizes. In this work, we have successfully carried out a dynamics study of an entire HIV capsid in full all-atom details. Our study reveals new insights into the dynamics of the N-terminal loops, the stabilizing role of the pentamers, and where the nucleotide transport may take place.

## Introduction

The recent breakthroughs in experimental technology for structure determination, especially in single-particle cryo-electron microscopy [2], have helped unveil many large structure assemblies at near atomic resolution for the first time. It is well recognized that a thorough knowledge of their dynamics can offer invaluable insight into their functional mechanisms and yet at the same time the enormous size of these systems poses a significant challenge to the computational simulations and analysis of their dynamics. Large scale computations comprising of millions of atoms are considered as one of the key problems by National Science Foundation’s (NSF) Molecular and Cellular Biosciences (MCB) program https://www.nsf.gov/funding/pgm_summ.jsp?pims_id=504858&org=MCB.

Normal mode analysis (NMA) [3–5] is a powerful tool for studying the intrinsic dynamics of biological assemblies. Mathematically, the core of all NMA computations involves solving a generalized eigenvalue problem of the Hessian matrix and the mass matrix. For extremely large assemblies, the source of the challenge in running NMA is the size of the Hessian matrix, whose dimension is in the same order as the number of atoms in the system. Precisely, for a system with *N* atoms, the Hessian matrix is of dimension 3*N* × 3*N*. For large systems with millions of atoms, it would take an extremely large amount of memory just to store the whole Hessian matrix even if a sparse matrix is used. To address this problem, two types of approaches have been developed. One is to use special eigenvalue solvers such as ARPACK [6] or order *N* technique [7, 8] that are designed to compute quickly a small number of eigenvalues and eigenvectors. Similar to standard eigenvalue solvers, this type of approaches still require knowledge of a full Hessian matrix (in the sparse matrix format), which can become severely limiting when dealing with extremely large systems such as HIV-1 capsid that has nearly 5 million atoms. The advantage of this type of approaches is that the accuracy is fully maintained and not compromised in any way.

The other type of approaches for solving the eigenvalue problem of extremely large systems is by projection. RTB [9] and BNM [10] are two well-known approaches of this kind. Lezon and co-workers [11], for example, successfully applied an RTB-based approach to compute the normal mode dynamics of HIV-1 capsid at a coarse-grained level. The advantage of projection-based methods is clear: it greatly reduces the size of the Hessian matrix. A major drawback of projection-based methods is the loss of accuracy, especially in normal modes of higher frequencies.

In our most recent work [1], we discovered a physical phenomenon that makes it possible to develop a new projection-based method that maintains all the advantages of projection-based methods and yet loses no or little accuracy. We discovered that the normal mode of a whole capsid at any given frequency *ω* is contributed nearly solely by vibrations of its individual capsomeres at around the same frequency, i.e., there is a sharp resonance between the vibrations of a whole capsid and those of its capsomeres.

Based on these observations, we were able to define a projection matrix **P**^(*i*)^ (1 ≤ *i* ≤ *m*, where *m* is the number of capsomeres) for each capsomere using the normal modes of the capsomere at a selected range of frequencies. The selection could be the modes below or a band of modes around a certain frequency [1]. For example, if there are *N* atoms in each capsomere and *k* modes are selected to represent **P**^(*i*)^, **P**^(*i*)^ will be a 3*N* × *k* matrix whose columns are the selected modes.

Given **P**^(*i*)^s, the projection matrix for the whole capsid is constructed as follows [1]:

$$\begin{array}{c} P=(\begin{array}{cccc} P^{\left(1\right)} & 0 & \cdots & 0\\ 0 & P^{\left(2\right)} & & 0\\ \vdots & & \ddots & \vdots \\ 0 & 0 & \cdots & P^{\left(m\right)} \end{array})\;, \end{array}$$ (1)

and the projected Hessian matrix is [1]:

$$\begin{array}{c} H_{s}=P^{⊤}HP, \end{array}$$ (2)

where **H** is the original Hessian matrix. **H**_*s*_ is now a much smaller matrix than **H** (assuming that *k* ≪ 3*N*) and thus is much easier to solve.

The present work is a continuation of our previous work on resonance and focuses on the following issues that were not addressed in the resonance paper [1]. Specifically, 1) we conduct a quantitative assessment of the quality of the modes produced by the BOSE model. The assessment is carried out using four capsid test cases whose normal modes (the benchmark) can be obtained directly from the original Hessian matrix. In addition to “cumulative overlap” that is commonly used to assess mode quality, we develop also a new measure called “degeneracy-based overlap” for assessing the quality of modes. 2) We develop an additional measure that can be used to predict the quality of modes for the case when benchmark modes are not available, which is often the case and is the very reason for the existence of projection-based methods. 3) We address the issue of block selection and its effect on the performance of the BOSE model. This is especially relevant for capsids of which the composition of capsomeres, which are used as panel blocks in BOSE, is not obvious from the literature. In such a situation, we show that the aforementioned mode quality predicting measure can be used to determine what is the best choice for panel blocks. 4) Lastly, we perform for the first time an all-atom normal mode analysis of an entire HIV-1 capsid, a system with nearly 5 million atoms.

## Methods

In this section, we first elaborate on the BOSE model, which is designed for normal mode computations of extremely large assemblies. It is followed by the presentation of several mode-quality assessment methods.

### The BOSE model

Recall that our aim here is to efficiently and accurately determine the normal modes of extremely large systems that have millions of atoms or more. We will focus on the low frequency normal modes in this work. The same method can be applied to obtain normal modes at other frequency ranges as well.

The key realization behind the BOSE model is that large structure assemblies are made up of many components, or copies of proteins of the same or similar structures. BOSE reduces the complexity of the normal mode computation by effectively modeling the elasticity of each block with a small, selected number of normal modes. For the sake of simplicity, we assume in the following that the system being studied is composed of identical protein chains, even though the method still works otherwise.

#### Panel block selection

Of the largest structures deposited in PDB [12], a large percentage of them are viral capsid structures (e.g., HIV-1 capsid [13]) or structures of bacterial microcompartments (e.g., [14]). The assembly units of these protein shells, called capsomeres, are the natural choice for panel blocks used in BOSE. The capsomeres often take the form of hexamers, pentamers, trimers, or dimers.

#### Modeling the elasticity of a panel block

In elastic network models, the elasticity of a protein is modeled by a network of springs, or an elastic network. The elasticity of such a network can be captured also in the form of a Hessian matrix [15]. The elasticity determines the dynamics of the system and manifests itself in the patterns of motions of the system and associated vibrational frequencies.

When computing the vibrational dynamics of a large system composed of many panel blocks, it is advantageous to focus only on the elasticity of each block that is most relevant to the vibrations of the whole system [1] at the frequency of interest. Our recent work revealed that there existed a strong resonance between the vibrations of a whole capsid and those of individual capsomeres [1]. That is, to reproduce the vibrations of a whole capsid at any given frequency *ω*, only a narrow band of normal modes at frequencies around *ω* of the capsomeres are needed. Therefore, to most efficiently compute the normal modes of a large capsid, we take the following steps. We first compute the normal modes of each panel block at around the desired frequency, such as the modes at the lowest frequency end. This step can be done by using ARPACK [6], which is designed for efficient computations of a small number of eigenvalues/eigenvectors. Next we model the elasticity of each block using these modes, for example, the lowest *l* modes. Using only *l* modes instead of using all the modes is advantageous since it can greatly reduce the size of the Hessian matrix (more details are given later) and thus make it possible to obtain normal modes of extremely large systems that otherwise would be impossible. Thus, in our model, the *i*-th block can elastically deform only along the directions of the chosen *l* normal modes $v_{1}^{\left(i\right)},\ldots ,v_{l}^{\left(i\right)}$, where $v_{j}^{\left(i\right)}$ is the *j*-th normal mode of the *i*-th block. Note that the superscript with parenthesis “^(*i*)^” represents the *i*-th block. The value *l* is a preset constant and specifies the degrees of elasticity of each block. The choice of *l* depends on what normal modes of the capsid are of interest to the user. This will be further discussed in the Results section.

We define the elasticity or the projection matrix **J**^(*i*)^ of the *i*-th block as the set of its first *l* normal modes, as follows, in a form of 3*n*_*i*_ × *l* matrix:

$$\begin{array}{c} J^{\left(i\right)}=(v_{1}^{\left(i\right)},v_{2}^{\left(i\right)},\cdots ,v_{l}^{\left(i\right)})\;. \end{array}$$ (3)

Note that the first six column vectors correspond to the rotational and translational degrees of freedom.

The elasticity matrix (or projection matrix) **J** of the whole system is defined by combining **J**^(1)^, …, **J**^(*m*)^ as follows, where *m* is the total number of blocks. Using the block matrix notation, we have:

$$\begin{array}{c} J=(\begin{array}{cccc} J^{\left(1\right)} & 0 & \cdots & 0\\ 0 & J^{\left(2\right)} & & 0\\ \vdots & & \ddots & \vdots \\ 0 & 0 & \cdots & J^{\left(m\right)} \end{array})\;, \end{array}$$ (4)

where **0** is a zero matrix.

#### Obtaining the dynamics of the whole system

The normal modes (of an individual block or the whole system) can be computed using either coarse-grained models or all-atom models. For simplicity, in the following derivation we concern not with the mass matrix. S1 Appendix presents the extra steps needed when the mass matrix is present.

Let **H** be the Hessian matrix in the Cartesian space. **H** can be written as a block matrix:

$$\begin{array}{c} H=(\begin{array}{ccccc} H_{1,1} & H_{1,2} & \cdots & H_{1,m-1} & H_{1,m}\\ \vdots & \vdots & \ddots & \vdots & \vdots \\ H_{i,1} & H_{i,2} & \cdots & H_{i,m-1} & H_{i,m}\\ \vdots & \vdots & \ddots & \vdots & \vdots \\ H_{m,1} & H_{m,2} & \cdots & H_{m,m-1} & H_{m,m} \end{array})\;, \end{array}$$ (5)

where *m* is the number of panel blocks (or capsomeres). **H**_*i,j*_ represents the interactions between panel blocks *i* and *j*. The reduced Hessian matrix $\tilde{H}$ is defined, using **J**, as follows:

$$\begin{array}{c} \tilde{H}=J^{⊤}HJ\;, \end{array}$$ (6)

where **J**^⊤^ is the transpose of **J**. In reality, there is no need to write down **H** or **J** explicitly since they may take too much memory. $\tilde{H}$ can be constructed block by block on the fly. Let,

$$\begin{array}{c} \tilde{H}=(\begin{array}{ccccc} {\tilde{H}}_{1,1} & {\tilde{H}}_{1,2} & \cdots & {\tilde{H}}_{1,m-1} & {\tilde{H}}_{1,m}\\ \vdots & \vdots & \ddots & \vdots & \vdots \\ {\tilde{H}}_{i,1} & {\tilde{H}}_{i,2} & \cdots & {\tilde{H}}_{i,m-1} & {\tilde{H}}_{i,m}\\ \vdots & \vdots & \ddots & \vdots & \vdots \\ {\tilde{H}}_{m,1} & {\tilde{H}}_{m,2} & \cdots & {\tilde{H}}_{m,m-1} & {\tilde{H}}_{m,m} \end{array})\;, \end{array}$$ (7)

where each block of $\tilde{H}$ can be constructed through:

$$\begin{array}{c} {\tilde{H}}_{i,j}={J^{\left(i\right)}}^{⊤}H_{i,j}J^{\left(j\right)}\;, \end{array}$$ (8)

Let ${\tilde{v}}_{i}$ and λ_*i*_ be the *i*-th eigenvector determined from $\tilde{H}$ and its corresponding eigenvalue, respectively. The *i*-th mode in Cartesian coordinate can be obtained as follows:

$$\begin{array}{c} v_{i}=J{\tilde{v}}_{i}\;. \end{array}$$ (9)

Note that ${\tilde{v}}_{i}$ is a column vector of length *lm* (which is the same as the number of columns in **J**) and takes the form:

$$\begin{array}{c} {\tilde{v}}_{i}={\left(c_{1,1}^{\left(i\right)},c_{1,2}^{\left(i\right)},\cdots ,c_{1,l}^{\left(i\right)},c_{2,1}^{\left(i\right)},c_{2,2}^{\left(i\right)},\cdots ,c_{2,l}^{\left(i\right)},\cdots ,c_{m,1}^{\left(i\right)},c_{m,2}^{\left(i\right)},\cdots ,c_{m,l}^{\left(i\right)}\right)}^{⊤}, \end{array}$$ (10)

where $c_{j,k}^{\left(i\right)}$ is a component of ${\tilde{v}}_{i}$. ${c_{j,k}^{\left(i\right)}}^{2}$ thus represents the contribution of the *k*^*th*^ mode of the *j*^*th*^ block in forming capsid mode ${\tilde{v}}_{i}$ and

$$\begin{array}{c} \sum_{j=1}^{l}\sum_{k=1}^{m}{c_{j,k}^{\left(i\right)}}^{2}=1 \end{array}$$ (11)

since ${\tilde{v}}_{i}$, as an eigenvector of $\tilde{H}$, is normalized.

### Assessing the quality of normal modes

To evaluate the quality of normal modes determined from the projected Hessian matrix, either that of BOSE or of RTB, the following two measurements are used. Both of them require a comparison with the benchmark normal modes computed directly from the original Hessian matrix. In the following, we use **v** to denote a mode determined by a projection-based method (BOSE or RTB), and **p** a mode determined from the original Hessian matrix (the benchmark modes).

#### Cumulative overlap (c-overlap)

Cumulative overlap (or c-overlap) measures the overlap between a mode and a group of modes: how well the mode is represented by the group of modes. The function c-ovlp(**p**, **v**_1..*n*_) calculates c-overlap between a mode **p** and a mode set **v**_1..*n*_ = {**v**_1_, …, **v**_*n*_} as follows:

$$\begin{array}{c} \text{c-ovlp}\left(p,v_{1..n}\right)={\left(\sum_{i=1}^{n}{\left(p\;\cdot \;v_{i}\right)}^{2}\right)}^{1/2}. \end{array}$$ (12)

c-ovlp(**p**, **v**_1..*n*_) indicates how well a benchmark mode **p** is covered by the subspace defined by the mode set **v**_1..*n*_. The higher c-ovlp(**p**, **v**_1..*n*_) is, the higher is the quality of the modes **v**_1..*n*_ as a group. Recall that **v**_1..*n*_ represents modes computed by BOSE or RTB, and **p** is one of the benchmark modes. Cumulative overlap thus defined assesses the quality of **v**_1..*n*_ as a group, not that of individual modes. To assess the latter, The following measure is used.

#### Degeneracy-based overlap (d-overlap)

We develop also a new assessment measure of the quality of individual modes computed by BOSE or RTB or other models. Note that each normal mode is associated with a vibrational frequency that can be determined from its eigenvalue. In [16], we demonstrated that normal modes with similar frequencies could easily mix together and become degenerate. Consequently, it is not meaningful to carry out a one-to-one comparison between a model mode (that of BOSE or RTB) and a benchmark mode. Rather, to take account of the effect of such a degeneracy, we define a degeneracy-based overlap, or d-ovlp(**v**, *P*), as the overlap between a model mode **v** and a narrow band of benchmark modes *P* at around the frequency of **v**, as follows:

$$\begin{array}{c} \text{d-ovlp}\left(v,P\right)={\left(\sum_{p\in f\left(v,P\right)}{\left(p\;\cdot \;v\right)}^{2}\right)}^{1/2}, \end{array}$$ (13)

where *f*(**v**, *P*) represents a narrow band of modes in *P* that have a similar frequencies to that of **v**, i.e.,

$$\begin{array}{c} f\left(v,P\right)=\left\{p|p\in P\;\text{and}\;\omega_{v}-\Delta \omega \leq \omega_{p}\leq \omega_{v}+\Delta \omega \right\}\;, \end{array}$$ (14)

where Δ*ω* is the degeneracy tolerance, and *ω*_**v**_ and *ω*_**p**_ are the frequencies of mode **v** and **p**, respectively. A degeneracy tolerance Δ*ω* = 3.0 cm^-1^ is used in this work. The choice of Δ*ω* is based on our recent study on resonance [1], which showed that a capsid mode of frequency *ω* is contributed mostly by block modes of frequencies [*ω* − Δ*ω*, *ω* + Δ*ω*] according to resonance, where Δ*ω* is 2–3 cm^-1^ for the all-atom sbNMA and 30-40 cm^-1^ for the coarse-grained ANM [1].

### Predict the quality of modes when no benchmark modes are available

The above mode quality assessment measures are still limited since in reality we generally don’t have the benchmark modes. The very reason for having the projection-based methods is that solving the eigenvalue problem of the original Hessian matrix is computationally prohibitive. Though we can assess the quality of BOSE modes on smaller systems for which benchmark modes are available and expect that the quality of BOSE modes remains the same by extrapolation, it is better to have a more direct way to predict the quality of modes.

In our resonance paper [1], we have shown that a capsid mode of frequency *ω* is contributed mostly by block modes at around the same frequency due to resonance. Consequently, to reproduce accurately a capsid mode of frequency *ω*, it is sufficient to include in the projection matrix only block modes of about the same frequency (see Eq (4)). Therefore, our first major step to ensure the quality of BOSE modes is to use only BOSE modes whose frequencies are within the range defined by the block modes, as modes outside the frequency range are not of reliable quality due to the principle of resonance.

Second, to quantify the cumulative contribution of a group of block modes to a given capsid mode ${\tilde{v}}_{i}$, we define block-mode cumulative square overlap (bmCSO) as follows:

$$\begin{array}{c} \text{bmCSO}\left({\tilde{v}}_{i},t\right)=\sum_{j=1}^{t}(\sum_{k=1}^{m}{c_{j,k}^{\left(i\right)}}^{2})\;, \end{array}$$ (15)

where $c_{j,k}^{\left(i\right)}$ is from Eq (10). The inner summation $\sum_{k=1}^{m}{c_{j,k}^{\left(i\right)}}^{2}$ represents the contribution to ${\tilde{v}}_{i}$ from the *j*^*th*^ modes of *all* the *m* panel blocks in the system. Summation $\sum_{j=1}^{t}$ denotes the cumulative contribution of the first *t* modes of all panel blocks. Clearly, $\text{bmCSO}({\tilde{v}}_{i},l)=1$ accordingly to Eq (11).

Later on in Results section, we will show that bmCSO strongly correlates with d-overlap and thus can be used as a predictor of the quality of BOSE modes. bmCSO is a variant of cumulative square overlap (CSO) that was used in [17].

### Panel block selection

As aforementioned, capsomeres are the natural choice for panel blocks to be used in BOSE. The capsomeres often take the form of hexamers, pentamers, trimers, or dimers. For most capsids, the composition of the capsomeres is clear. Most capsomeres are so stable that they exist in isolation. For a few other capsids, it is not clear even from the literature what is the composition of the capsomeres: are they pentamers, trimers, or dimers? Fortunately, as will be shown in Results section, our mode quality assessment measure is capable of indicating what is the best choice for panel blocks, especially when it is not obvious.

### Structure preparation

In this work, we use four small capsids for benchmark tests before applying the BOSE model to the large HIV-1 capsid. The benchmark structures are prepared in the following way:

1. Obtain structure coordinates from PDB (e.g., 4v4m.cif);
2. Select the first chain as the first asymmetric unit (or ASU);
3. Use VMD’s [18] psfgen to fill in the missing hydrogen atoms;
4. Use NAMD [19] to run an energy minimization while fixing positions of all heavy atoms;
5. Compute the 5-fold symmetry axis (**k**_5_) and a 3-fold symmetry axis (**k**_3_) near the first ASU;
6. Using the first ASU, **k**_5_, and **k**_3_, construct a fully symmetric icosahedral complex.

The HIV-1 capsid structure is prepared by taking steps 1, 3, and 4. The structures used by ANM are obtained by keeping only the *C*^*α*^ atoms.

### NMA model used and mean-square fluctuations

In our experiments, spring-based Normal Mode Analysis (sbNMA) [20] is used in all normal mode computations.

#### Spring-based normal mode analysis (sbNMA)

In Ref. [20], we developed a spring-based NMA (sbNMA) that closely resembles the classical NMA and yet requires no energy minimization. sbNMA is an all-atom model and uses an all-atom force field, such as CHARMM [21], AMBER [22], etc. The classical NMA Hessian matrix **H**^NMA^ can be written as a summation of two groups of terms: the spring-constant-based terms **H**^spr^ and the force/torque-based terms **H**^frc^ (proportional to the inter-atomic force or torques) [20, 23], i.e.,

$$\begin{array}{c} H^{\text{NMA}}=H^{\text{spr}}+H^{\text{frc}}. \end{array}$$ (16)

The contribution of **H**^frc^ was shown to be much smaller than the spring-constant-based term, accounting for only 10%. By keeping only the spring-constant-based terms, sbNMA still resembles closely the classical NMA and is able to yield high quality vibrational modes. Furthermore, it requires no energy minimization since the force/torque-based terms are removed. The sbNMA Hessian matrix is also highly sparse, making it easy to use with the proposed BOSE model on large structures. sbNMA was successfully applied to determine the functional dynamics of large protein complexes such as GroEL/GroES [24] and p97 [25], and the vibrational spectra of globular proteins [26]. In this work, sbNMA is used to compute the dynamics of HIV-1 capsid.

#### Mean-square fluctuation (MSF)

Mean-square fluctuations (MSFs) have often been used to evaluate computational models by comparing them with experimental B-factors. In our study, the mean-square fluctuation of *i*-th atom from the first *k* low frequency modes is calculated as follows [27]:

$$\begin{array}{c} B_{i}=\frac{8\pi^{2}k_{\text{B}}T}{3}\sum_{j=7}^{k}\frac{{\left[v_{j}\right]}_{i}^{⊤}{\left[v_{j}\right]}_{i}}{\lambda_{j}}\;, \end{array}$$ (17)

where *k*_B_ is the Boltzmann constant, *T* is the temperature in Kelvin, **v**_*j*_ and λ_*j*_ are *j*-th mode and its corresponding eigenvalue, respectively, and [**v**_*j*_]_*i*_ is the 3 × 1 displacement vector of the *i*-th atom in mode **v**_*j*_. In our study, the room temperature (300K) is used for *T*.

## Results

In this section, we first assess the quality of BOSE modes on a few test systems and compare it with the mode quality of the well-known RTB model. The comparison shows BOSE modes are of significantly higher quality, demonstrating the importance of having a proper modeling of the elasticity of each panel block. Modeling each capsomere using a few rigid blocks is shown to be insufficient. Moreover, most capsomeres appear to be a continuous body and rigid block partitions often seem arbitrary.

In the second half of this section, we apply BOSE to an extremely large system that has nearly 5 million atoms: HIV-1 capsid. Our all-atom NMA study of this extremely large system reveals some novel insights about its dynamics that may be beyond the reach of molecular dynamics simulations [28–30].

### Test cases

We use four capsids as test cases to evaluate the quality of BOSE modes. The four capsids are: capsid of Satellite Tobacco Necrosis Virus (STNV, pdb-id: 4V4M) [31], capsid of Sesbania mosaic virus (SeMV, pdb-id: 4Y5Z) [32], a mutant structure of the capsid of Grouper nervous necrosis virus (GNNV, pdb-id: 4RFT) [33], and capsid of a lumazine synthase from the thermophilic bacterium Aquifex aeolicus (AaLS, pdb-id: 5MPP) [34]. The four capsids all have icosahedral symmetry.

### Assessing the quality of modes of BOSE and RTB

BOSE and RTB are both projection-based methods that reduce the size of the original Hessian matrix through restricting the motion space of structural building blocks, which can be either protein chains, capsomeres, or groups of residues. RTB restricts the motion of each building block to only rigid body motions. BOSE treats each building block still as a flexible unit, by modeling its elasticity using a selected subset of its normal modes. BOSE thus restricts the motions of each building block by allowing only vibrations within a certain frequency range. The allowed vibrations or normal modes define the selected elasticity of the block (which is a capsomere).

To have a fair comparison between BOSE and RTB, we let the two models have the same degrees of freedom for each capsomere. Consequently, the size of their reduced Hessian matrices are the same. The accuracy of either model is measured by comparing its modes with the benchmark modes determined from the original Hessian matrix.

#### Experiment setup

Given a structure assembly, we first compute its normal modes directly from the original Hessian matrix, and then compute the modes using RTB and BOSE. Taking the STNV capsid (pdb-id: 4V4M) for example, which is composed of 12 pentamers and thus 60 protein chains and 184 residues (2,857 atoms) in each chain, we perform the following operations:

1. Determine the full-size sbNMA Hessian matrix **H** and compute all the modes directly from **H**. Since there are *n* = 2,857 * 60 = 171,420 atoms, the dimension of **H** is 514,260 × 514,260. To solve the eigenvalue problem of **H**, we apply group theory to take advantage of the capsid’s icosahedral symmetry [35–37]. As a result, the largest matrix needed to be solved is 12 times smaller [35–37].
2. To obtain the RTB modes, we first determine the rigid blocks. One way to select the rigid blocks of each protein chain is by observing the cross correlation patterns in the low frequency modes. There is often not a clear cut when deciding the boundary of a block and consequently the resulting selection is somewhat arbitrary. Once we have the rigid blocks, we can compute the projection matrix **P**. **P** restricts the motion of each block to rigid-body motions only. Lastly, the RTB Hessian matrix is **H**_RTB_ = **P**^⊤^**HP**. We set the number of blocks per protein chain to be 5. The ranges of residues of the five rigid blocks selected for 4V4M are: 12-24, 25-60, 61-100, 101-147, and 148-195. Consequently, there are 25 rigid blocks per pentamer (capsomere), or 300 rigid blocks for the whole capsid. The total degrees of freedom for the whole system is 300 * 6 = 1,800. The dimension of **H**_RTB_ is thus 1,800 × 1,800, whose size is about 286 times smaller than the original Hessian matrix **H**.
3. For the BOSE model, there is no need for picking rigid blocks. Instead, for each capsomere, we apply sbNMA to compute its first 150 normal modes (including the first six rigid body modes) and use them to model the elasticity of each capsomere. The resulting reduced Hessian matrix **H**_BOSE_ also has the same dimension of 1,800 × 1,800.

Note that in BOSE, *l* is set to be 150 in Eq (3), i.e., 150 normal modes are selected per capsomere. This guarantees that both **H**_BOSE_ and **H**_RTB_ have the same dimension (1,800 × 1,800) after projection. This way we can have a fair comparison between the BOSE model and the RTB model.

#### Quality comparison between BOSE and RTB

Once we have all the modes, we assess the quality of modes of RTB or BOSE using cumulative overlap (c-overlap) and degeneracy-based overlap (d-overlap), using sbNMA modes as the benchmark. Both BOSE and RTB use a reduced Hessian matrix, and both produce 1,800 modes. We now compare these 1,800 modes with the first 1,800 modes of sbNMA.

**Fig 1:**
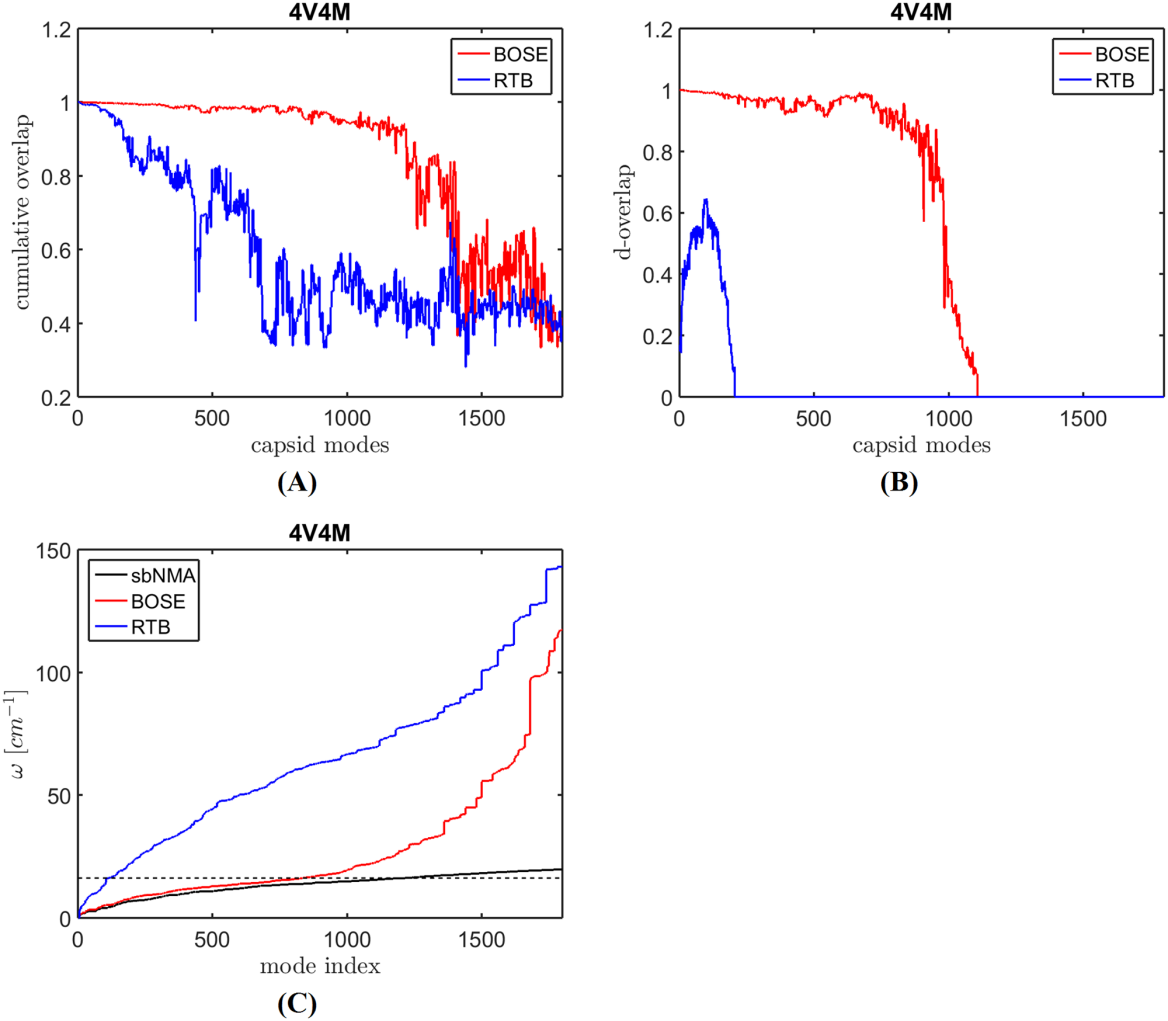
Mode quality comparison between RTB and BOSE. (A) The c-overlaps (or cumulative overlaps, see Methods section) between sbNMA benchmark modes and all the modes of BOSE or RTB. (B) The d-overlaps (or degeneracy-based overlap, see Methods section) between a BOSE or RTB mode and a narrow band of benchmark modes after considering degeneracy. (C) shows the frequencies of the modes generated by sbNMA (the benchmark), BOSE, and RTB. The dashed line marks the upper limit of the panel mode frequencies. It marks a frequency threshold above which BOSE modes are no longer reliable due to resonance [1]. Under this threshold value, The BOSE modes’ frequencies match nearly perfectly with those of sbNMA, the benchmark. The frequencies of RTB modes are clearly too high.

Fig 1(A) shows the cumulative overlaps (c-ovlp in Eq (12)) between an sbNMA mode and all the modes of RTB or BOSE. The figure shows that BOSE modes cover nearly fully the low-frequency sbNMA mode space, while RTB modes cover a significantly less amount. Fig 1(B) shows the degeneracy overlap (d-overlap) between sbNMA and RTB or BOSE. In the figure, the d-overlaps are determined with a frequency tolerance of Δ*ω* = 3 cm^-1^ (Eqs (13) and (14)). The figure shows that BOSE modes are highly similar to sbNMA modes. In contrast, the d-overlap for RTB is significantly worse. It indicates that RTB modes in general do not have a matching sbNMA mode in a similar frequency range.

Fig 1(C) shows the frequencies of the modes generated by sbNMA (the benchmark), BOSE, and RTB. The dashed line marks the upper limit of the panel mode frequencies. It marks a *frequency threshold* above which BOSE modes are no longer reliable due to resonance [1]. Under this threshold value, the BOSE model produces modes whose frequencies match nearly perfectly with those of sbNMA (the benchmark). The frequencies of RTB modes are clearly too high.

In summary, results in Fig 1 clearly indicate that BOSE modes are of high quality within the frequency range modeled by the capsomeres and BOSE is significantly better than RTB in preserving both the normal modes and the vibrational frequency spectrum.

**Fig 2:**
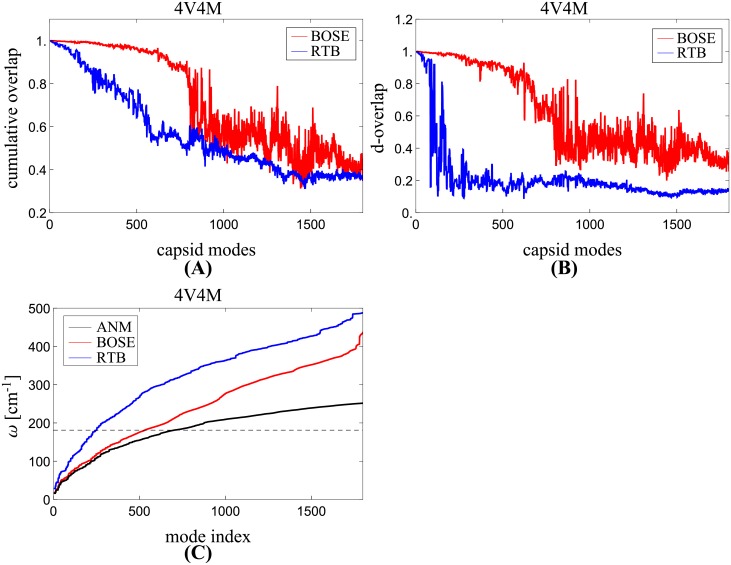
Mode quality comparison between RTB and BOSE using ANM as the benchmark. This figure is the same as Fig 1 except that ANM is used to generate benchmark modes instead of sbNMA. (A) The c-overlaps between ANM benchmark modes and all the modes of BOSE or RTB. (B) The d-overlaps between a BOSE or RTB mode and a narrow band of benchmark modes after considering degeneracy. (C) The frequencies of the modes generated by ANM (the benchmark), BOSE, and RTB. The dashed line marks the upper limit of the panel mode frequencies.

To show the results obtained above (Fig 1) are independent of the benchmark model used, we repeat the same experiments but use the coarse-grained ANM [15] model as the benchmark instead. Under ANM, the whole capsid of STNV (pdb-id: 4V4M) is modeled as a network of 11,040 nodes (one node per residue), which is much smaller in size than the corresponding all-atom model that has 171,420 atoms. Since the system is small enough, we first apply ANM directly to obtain the exact ANM normal modes of the whole system. These modes are used as the benchmark. Next, following the procedure described above, we obtain two more sets of normal modes using projection methods RTB and BOSE. By comparing the approximate solutions obtained from RTB or BOSE with the exact benchmark ANM modes, we can assess which projection method is better. Fig 2 shows the results, which are the same as Fig 1 except that ANM is used to generate the benchmark modes instead of sbNMA and that d-overlap in Fig 2(B) is computed with a larger frequency tolerance of Δ*ω* = 40 cm^-1^ suited for ANM [1]. Overall, the results in Fig 2 have a similar trend as those in Fig 1, both showing that BOSE modes are significantly better than RTB modes.

### Predicting the quality of BOSE modes when benchmark modes are unavailable

In this section, we present ways to predict the quality of BOSE modes when benchmark modes are not available. This will be the case when applying BOSE to compute the normal modes of new capsids, especially those that are so large that it is infeasible to compute their normal modes without employing a projection-based method. For such systems, we cannot apply cumulative overlaps or degeneracy-based overlaps to assess the mode quality since the benchmark modes are not available.

**Fig 3:**
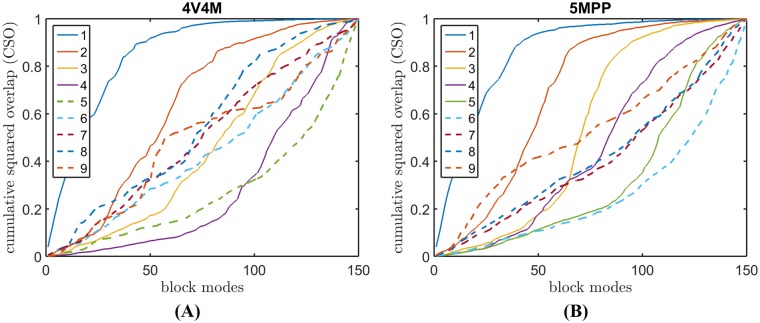
Cumulative contributions of block modes to the normal modes of two whole capsids: STNV(pdb-id: 4V4M) [31] (A) and AaLS (pdb-id: 5MPP) [34] (B). All the 1800 modes of the capsids are equally divided into 9 groups. Each curve represents the average *bmCSO* of the modes within that group.

Fig 3 shows the cumulative contributions of block modes to the normal modes of two whole capsids: STNV(pdb-id: 4V4M) [31] and AaLS (pdb-id: 5MPP) [34]. In the figure, all the 1800 modes of the capsid are equally divided into nine groups. Each curve represents the average *bmCSO* (see its definition in Methods section) of the modes within that group. The solid line curves represent capsid modes whose frequencies are within the range of the frequencies of the block modes. For these modes, we have confidence of their quality due to the principle of resonance [1]. The remaining groups of modes whose frequencies are out of range are drawn in dashed lines. The quality of these modes are unreliable.

**Fig 4:**
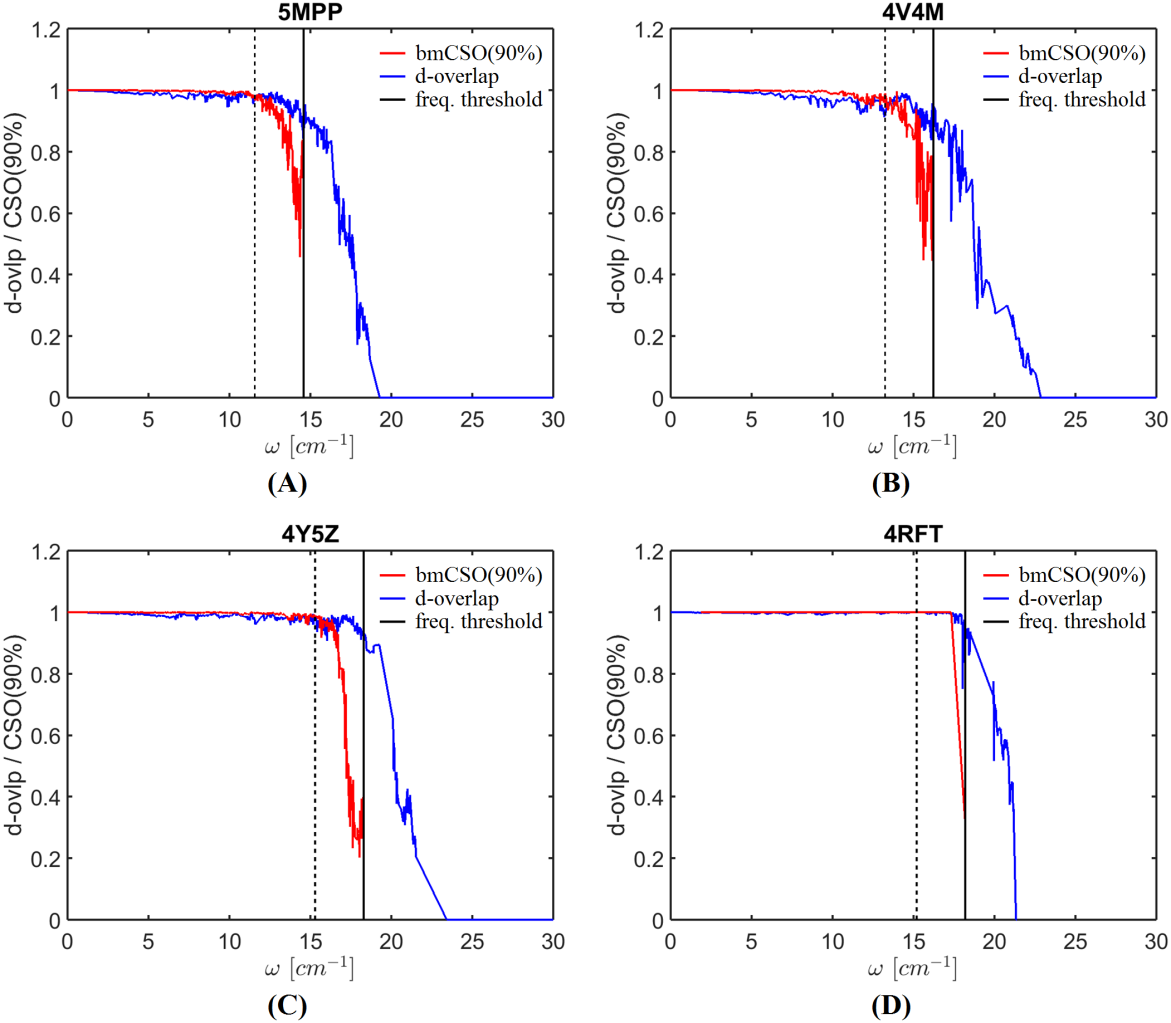
The close match between bmCSO and degeneracy-based overlaps of the BOSE modes at frequency *ω* of four whole capsids: AaLS (pdb-id: 5MPP) [34] (A), STNV(pdb-id: 4V4M) [31] (B), SeMV (pdb-id: 4Y5Z) [32] (C) and GNNV (pdb-id: 4RFT) [33] (D). The red line represents the block-mode cumulative squared overlap (bmCSO) when the first 90% of the block modes are used, or bmCSO(90%). The blue line shows the d-overlap of the BOSE modes. The black solid vertical line marks the location of the frequency upper limit of the block modes. The dashed line marks where it is 3 cm^-1^ below the frequency upper limit.

The bmCSO plot can be used to predict the quality of BOSE modes. To demonstrate this, we plot in Fig 4 bmCSO and degeneracy-based overlap (d-overlap) for all four capsids. The d-overlap assesses the quality of modes at different frequencies (abscissa axis). The red line shows the block-mode cumulative square overlap (bmCSO) when the first 90% of the block modes are used, or bmCSO(90%). The blue line shows the degeneracy-based overlap of the BOSE modes. A d-overlap value close to 1 means high quality. The black solid vertical line marks the frequency upper limit of the panel block modes. It marks a frequency threshold above which capsid modes are no longer of good quality due to resonance, as seen in the sharp drop in the blue lines. Thus only modes below the frequency threshold (i.e., to the left of the vertical line) are of interest. Under this frequency threshold, bmCSO(90%) (red line) matches closely with d-overlap (blue line), implying that both the frequency threshold and bmCSO(90%) are good indicators of mode quality.

Notice that in the zone between the dashed vertical line, which is 3 cm^-1^ to the left of the solid vertical line, and the solid vertical line itself, bmCSO(90%) starts to drop significantly while d-overlap remains fairly high. The reason is that a capsid mode of frequency *ω* is contributed mostly by block modes of frequencies [*ω* − Δ*ω*, *ω* + Δ*ω*] where Δ*ω* is 3 cm^-1^ according to resonance [1]. The gap between the solid and dashed lines thus represents a “twilight” zone: the quality of modes in this frequency range is still fairly good according to d-overlap though it is not evident from the bmCSO (90%) measure.

In summary, when computing the normal modes of a new capsid using BOSE, we have two ways to assess the quality of the modes according to Fig 4. One is to simply use the frequency threshold, the vertical solid line in Fig 4: normal modes below this frequency all have a high d-overlap value. The other is to use bmCSO(90%): normal modes with a large bmCSO(90%) value also have a high d-overlap value.

### Choosing the right panel blocks

We investigate also the effect of panel block selection on the mode quality. This is especially necessary for cases when the choice of the capsomeres is not obvious. In the following, we consider two capsids: one is the capsid of Sesbania mosaic virus (SeMV, pdb-id: 4Y5Z) [32] and the other is a mutant structure of the capsid of Grouper nervous necrosis virus (GNNV, pdb-id: 4RFT) [33]. From the literature where the structures of these two capsids were first reported [32, 33], it is not entirely clear what are the capsomeres of these capsids: are they pentamers or trimers or something else?

In the following, for both capsids, two different choices of panels are tested: i) using trimers as panel blocks and 90 modes per panel block; ii) using pentamers as panel blocks and 150 modes per panel block. In both selection schemes, a total of 1,800 BOSE modes are generated for the whole system and compared with the benchmark modes of sbNMA.

**Fig 5:**
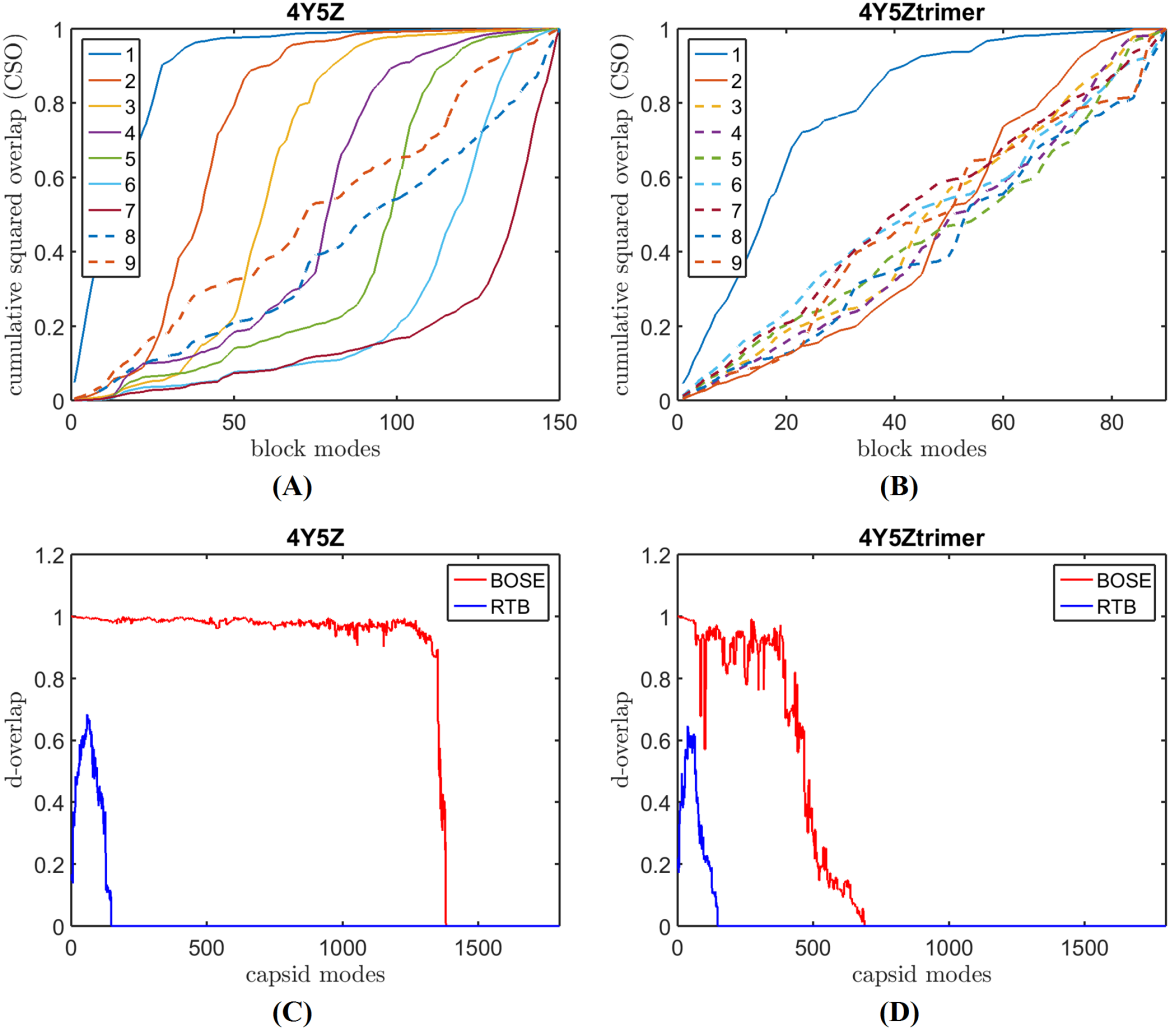
The effect of panel block selection on the mode quality of SeMV capsid. (A) and (C) show the bmCSO and d-overlap plots of SeMV capsid (pdb-id: 4Y5Z) respectively when pentamers are used as panel blocks. In contrast, (B) and (D) show the same plots except that trimers are used as panel blocks.

**Fig 6:**
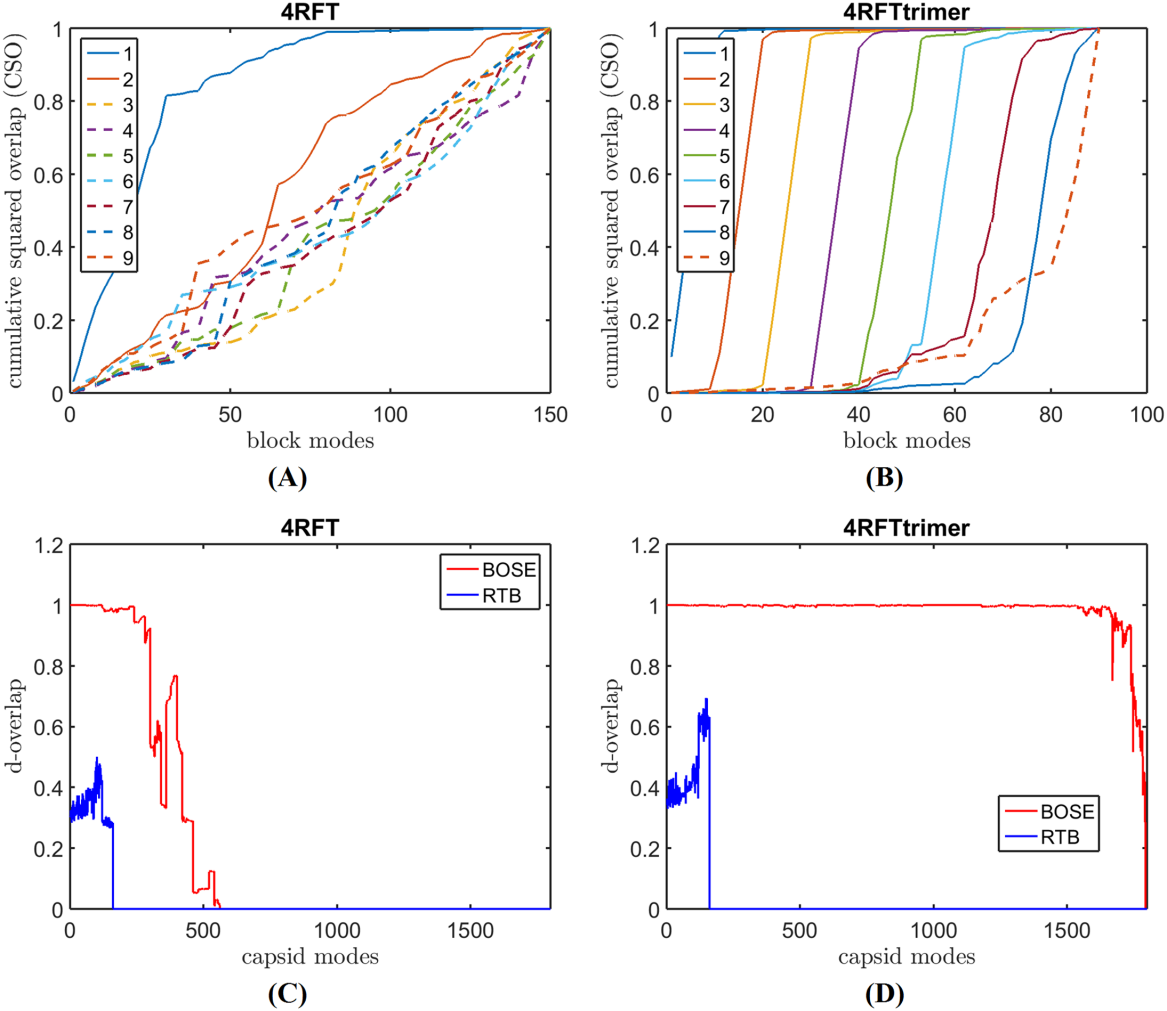
The effect of panel block selection on the mode quality of GNNV capsid. (A) and (C) show the bmCSO and d-overlap plots of GNNV capsid (pdb-id: 4RFT) respectively when pentamers are used as panel blocks. In contrast, (B) and (D) show the same plots except that trimers are used as panel blocks.

Fig 5 shows bmCSO and d-overlap plots of SeMV capsid when pentamers are used as panel blocks (panels (A) and (C)) and when trimers are used as panel blocks (panels (B) and (D)). The mode quality is significantly better when pentamers are used. The same plots are repeated in Fig 6 for GNNV capsid, for which the opposite is true: the mode quality is significantly better when trimers are used as panel blocks. In both cases, even without seeing the d-overlap plots that shows the quality of the modes but cannot be computed without the benchmark modes, the bmCSO plots clearly reveal what panel choices are better. The right choice of panel blocks produces not only significantly higher bmCSO values but also more modes of reliable quality below the frequency threshold, i.e., more solid lines and fewer dashed lines (see panels (A) and (B) in Figs 5 and 6).

### Comparisons of computational costs between BOSE and RTB

Table 1 lists the computational costs of BOSE, RTB, or sbNMA. The sbNMA Hessian matrix without symmetricity consideration would take more than 2 Tb memory space, which is too large for most computer systems. However, by applying group theory and taking advantage of the inherent icosahedral symmetry [35–37], the Hessian matrix can be reduced to 10–15 Gb and normal modes can be obtained without losing any accuracy. On the other hand, both BOSE and RTB use a significantly less amount of memory. BOSE uses about 30% more computational time than RTB. The extra time is spent on computing the normal modes of the capsomeres.

**Table 1:** CPU times and memory usages in running all-atom sbNMA, BOSE, and RTB.

	number of atoms per capsomere / capsid	BOSE (Mem: 233 Mb^a^) CPU time	RTB (Mem: 233 Mb^a^) CPU time	sbNMA (≈ 2Tb w/o symmetry)^b^	
				Memory^c^	CPU time^c^
4V4M	2,857 / 171,420	6,056 sec	4,877 sec	14.7 Gb	31,000 sec
5MPP	2,378 / 142,680	4,160 sec	3,212 sec	10.2 Gb	26,800 sec
4Y5Z	2,849 / 170,940	6,501 sec	5,125 sec	14.6 Gb	32,564 sec
4RFT	2,499 / 149,940	4,508 sec	3,646 sec	11.2 Gb	28,068 sec

^*a*^The reduced Hessian matrix size of BOSE or RTB is 233 Mb;

^*b*^The sbNMA Hessian matrix without symmetry consideration takes about 2 Tb;

^*c*^The memory usage and running time for sbNMA with symmetry consideration.

### Application to the HIV-1 capsid

In this section, we apply the BOSE model to study the normal mode dynamics of an extremely large system in atomic details, the HIV-1 capsid. The HIV-1 capsid is a large structure with a molecular mass of 35 MDa and has nearly 5 million atoms. Because of its extremely large size, all-atom normal mode computations of this assembly are prohibitive on most computer systems. Our projection-based BOSE model allows us to perform all-atom normal mode computations of this large assembly for the first time. Our normal mode computations reveal in atomic details the intrinsic motion patterns of this large structure, particularly the dynamics of the pentamers, N-terminal loops of the capsid proteins, and hexamer pores.

#### The HIV-1 capsid structure

**Fig 7:**
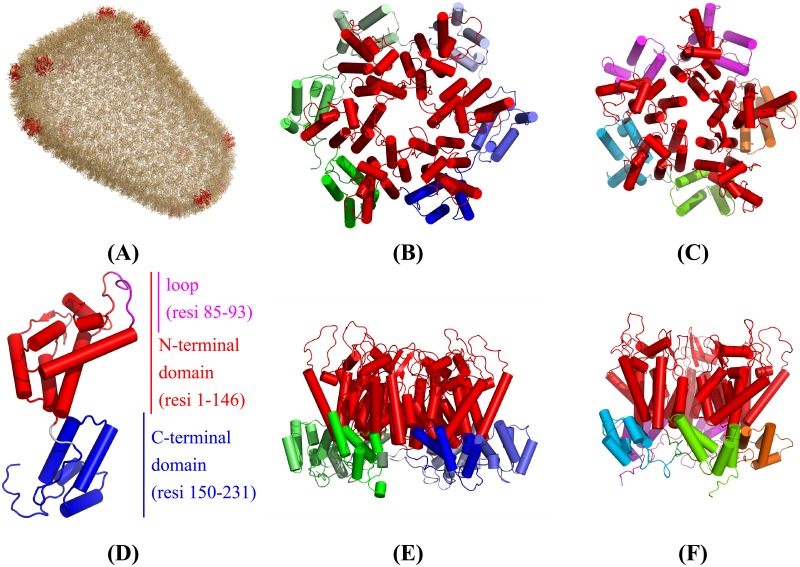
Structures of HIV-1 capsid, hexamer, pentamer, and capsid protein. (A) The whole structure of an HIV-1 capsid. Hexamers and pentamers are colored light-orange and red, respectively. (D) Structure of the capsid protein. A hexamer structure is shown in (B) the top view and (E) the front view. A pentamer structure is shown in (C) the top view and (F) the front view. All N-terminal domains in (B)–(F) are colored red.

HIV-1 capsid is the protein shell of HIV-1 virus and is made up of over a 1,000 copies of a single capsid protein (CP) in the form of hexamers and pentamers. Fig 7(A) shows the whole structure of a HIV-1 capsid (pdb-id: 3J3Q), where hexamers and pentamers are colored light-orange and red, respectively. Fig 7(D) shows the structure of the capsid protein (CP) with its 231 residues. Each capsid protein is composed of two domains, N-terminal domain and C-terminal domain, connected by a short linker. In the figure, the N-terminal domain (or NTD, residues 1–146) that is exposed on the outer surface of the capsid is colored red, while the C-terminal domain (or CTD, residues 150–231) that forms the inner surface of the capsid shell is colored blue. The loop (residues 85–93) in the N-terminal domain is colored pink. Fig 7(B) and 7(E) show the structure of a hexamer in the top and front views, respectively. Fig 7(C) and 7(F) show the structure of a pentamer in the top and front views, respectively. In HIV-1 capsid structure, the positions of pentamers determine the shape of the assembly and 12 pentamers are needed to form a closed cone [38]. In Fig 7(B)–7(F), all N-terminal domains are colored red.

#### Panel block selection for HIV-1 capsid

Performing the traditional normal mode analysis of the HIV-1 capsid structure is prohibitive on most computer systems due to its large size. It has over 300,000 residues and nearly 5 million atoms. Even for a coarse-grained *C*^*α*^-based model, it would require over 600 Gb memory space just to store its Hessian matrix (if a sparse matrix format is not used) and a similar amount of memory to store all the modes (if all the modes are needed). Because of this high memory requirement, Bergman and Lezon simplified their coarse-grained model even further by employing the RTB model and represented each capsid protein with 7 rigid blocks [11]. Here, we apply the BOSE model to obtain the normal modes of this extremely large capsid in atomic details. To preserve the dynamics in all-atom accuracy, we use spring-based NMA (sbNMA) [20] to compute the normal modes, as we did with the test cases. To apply BOSE, we first model each capsomere (hexamer or pentamer) as a panel block. All together, there are 228 panel blocks on the whole capsid. Next, sbNMA [20] is applied to each panel block to construct an accurate all-atom Hessian matrix and from which the first 150 lowest frequency modes are obtained. These 150 low frequency modes are then used to represent the selected elasticity of each panel block. Since there are 228 panel blocks, the projection subspace (see Eq (6)) has a dimension of 150 * 228 = 34,200. The reduced Hessian matrix $\tilde{H}$ in Eq (6) thus has a dimension of 34,200 × 34,200 and occupies about 8.7 Gb memory space. We choose to use 150 modes per panel block for two reasons. First, with 150 modes per capsomere, it is still feasible to compute the BOSE modes of the entire HIV-1 capsid using our workstation that has 64 Gb memory. Second, the first 150 modes of the capsomeres represent the low frequency modes in the range of [0, 8.8 cm^-1^]. Using 150 modes allows us to obtain the normal modes of the whole HIV-1 in a similar frequency range, which is enough for our analysis of the low frequency normal mode dynamics of the capsid to be shown next. Should one desire to obtain the normal modes of the whole capsid in a different frequency range, the normal modes of the capsomeres should be selected accordingly.

**Fig 8:**
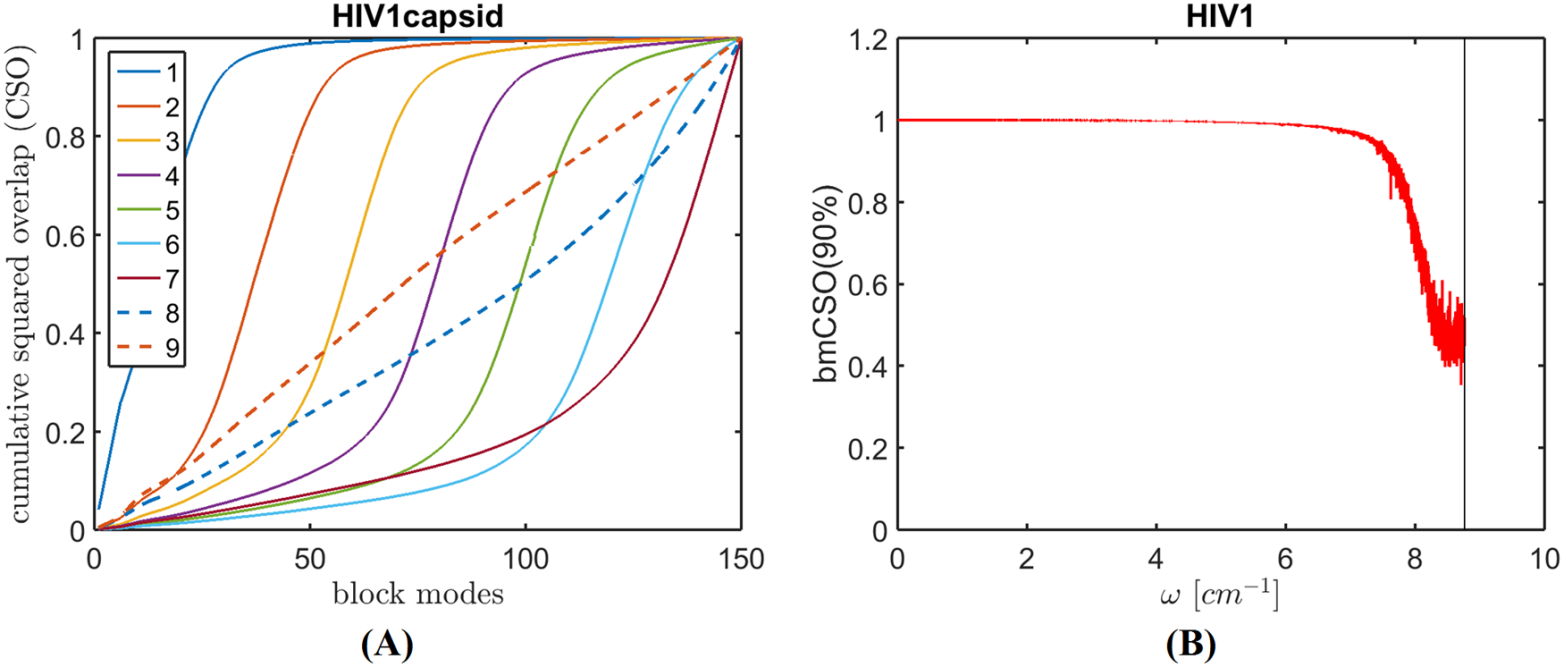
Cumulative contributions of the block modes to the modes of HIV-1 capsid. (A) The block-mode cumulative square overlap (bmCSO) plot. (B) bmCSO(90%) for modes whose frequencies are below the threshold (marked by the vertical line).

To assess the quality of the BOSE modes of this large capsid, the bmCSO plot is drawn in Fig 8(A). As in Fig 3, all the modes of the capsid are equally divided into nine groups. The solid lines represent capsid modes whose frequencies are below the threshold (the frequency upper limit defined by the block modes), while the dashed lines represent capsid modes whose frequencies are above the threshold. Fig 8(A) shows what block modes contribute to the different groups of capsid modes. Groups for which bmCSO reaches nearly 1 before all the block modes are considered are well reproduced by the block modes and are thus of good quality. Fig 8(B) shows bmCSO(90%) of the BOSE modes versus their frequencies. The vertical solid line marks the frequency upper limit of the block modes. As aforementioned, both bmCSO(90%) are the frequency threshold (upper limit) are good indicators of mode quality, as degeneracy-based overlap or d-overlap takes a high value (nearly 1) for the modes below this frequency threshold and bmCSO(90%) strongly correlates with d-overlap. Fig 8(B) shows the bmCSO(90%) value is nearly 1 for most of the modes below the frequency threshold, further confirming that the modes below 8.8 cm^-1^ (the frequency threshold) are of high quality.

#### The low frequency spectrum of HIV-1 capsid

We next examine the frequency spectrum of all 34,200 normal modes obtained from the BOSE model. As will be shown in the following, these modes reveal the low frequency motion patterns of HIV-1 capsid.

**Fig 9:**
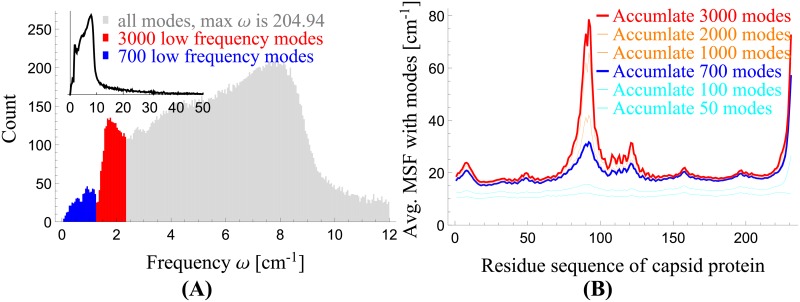
The vibrational frequency spectrum of the HIV-1 capsid and the MSFs of the capsid proteins. (A) The vibrational frequency spectrum as computed by BOSE, with the first two mode groups highlighted in blue and red. (B) MSFs determined using the first 700 and 3,000 modes are shown in blue and red, respectively, whose corresponding frequency ranges are given in (A).

Fig 9(A) shows the frequency spectrum. The spectrum can be roughly divided into three groups according to the range of frequencies: 0–1.3 cm^-1^ (the first 700 modes, in blue), 1.3–2.3 cm^-1^ (701st–3,000th modes, in orange), and 2.3 cm^-1^ and higher (3,001st and higher, in gray). Since the lowest frequency modes tend to be of the most interest, we focus our attention on the first two groups of modes only: 0–1.3 cm^-1^ in blue, and 1.3–2.3 cm^-1^ in red. The inset of Fig 9(A) shows the same spectrum up to 50 cm^-1^.

Fig 9(B) shows the mean-square fluctuations (MSFs) of the capsid proteins, computed using only the first 3,000 low-frequency modes (the first two groups of modes mentioned above) and averaged over all 1,356 chains. In the figure, the blue (red) line shows the average MSF determined using the first 700 (3,000) modes, which corresponds to the blue (red) region in Fig 9(A). In the following two sections, we will discuss the dynamic roles of these two mode groups in more details.

#### Dynamics present in the first and second mode groups

Our results reveal that motions of the pentamers in the first mode group help stabilize the HIV-1 capsid structure, as indicated by their suppressed vibration (or mobility) at both hemispherical ends, and that the boundary between the first and second mode groups (at about 1.3 cm^-1^) clearly marks a transition point from global motions in the first mode group to more localized motions in the second mode group, particularly motions of the NTD loops. Additionally, our results suggest that nucleotide transport may take place mostly at hexamers near pentamers.

#### The role of pentamers in the first mode group

Structural studies show that pentamers incur sharp curvatures while forming hemispherical ends of the capsule-shaped HIV-1 capsid surface [38, 39]. However, the dynamic role of the pentamers has not been thoroughly examined. Our study shows that one of the roles of the pentamers is to stabilize the HIV-1 capsid structure through suppressing the fluctuation dynamics at both hemispherical ends.

**Fig 10:**
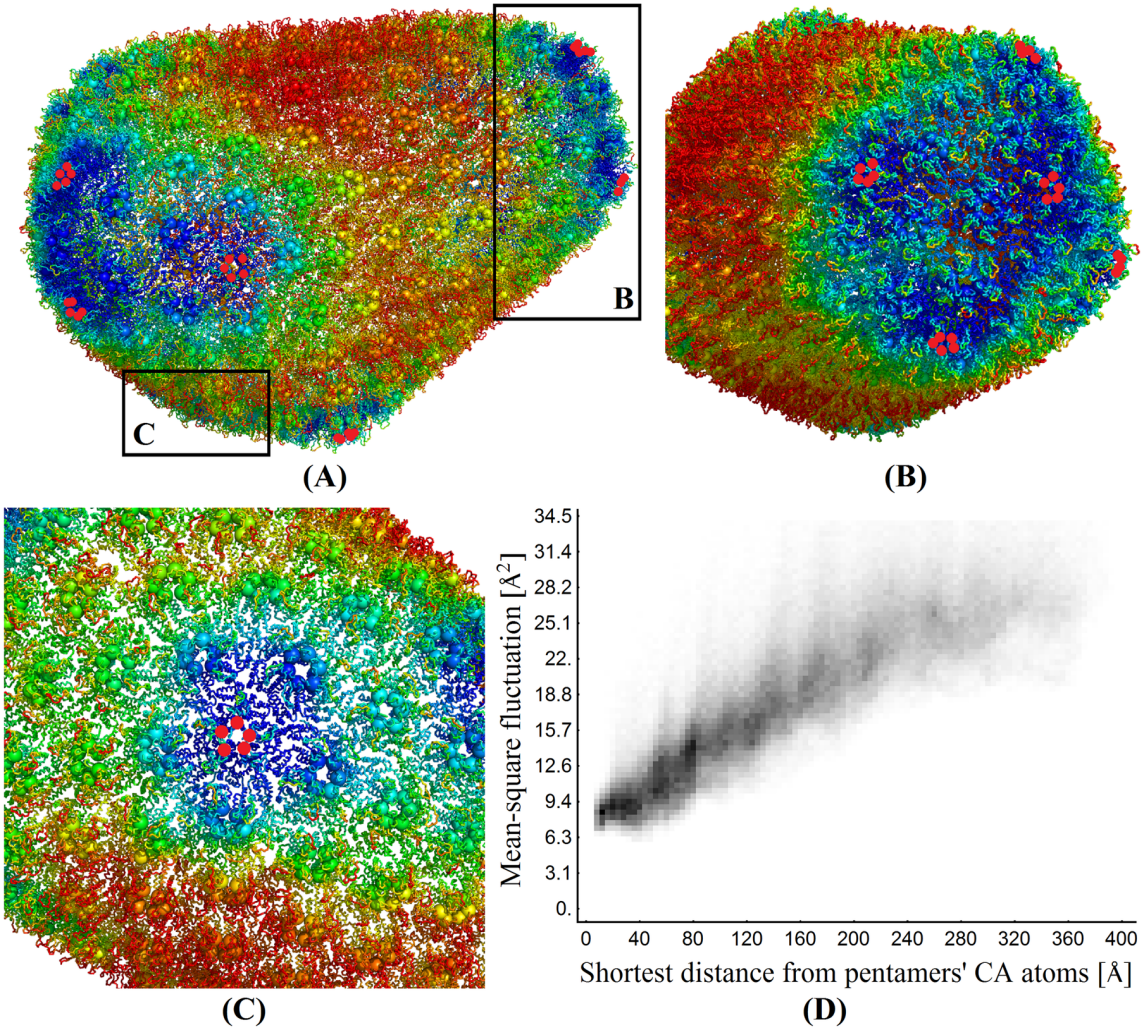
Global fluctuation dynamics near pentamers as determined using the first group of modes. (A)–(C) show the HIV-1 capsid structure in different orientations, color-coded by MSFs. Dark blue and red colors represent MSF smaller than 7.1 Å^2^ and larger than 31.4 Å^2^, respectively. (D) The correlation between a hexamer’s MSFs and its distance to the closest pentamer. The grayscale of a pixel represents the number of *C*^*α*^ atoms that have that MSF and distance, ranging from zero (in white) to as many as 528 (in black).

Fig 10(A)–10(C) show the HIV-1 capsid structure color-coded by MSFs: MSF of dark blue regions are smaller than 7.1 Å^2^, and MSF of red regions are larger than 31.4 Å^2^. MSFs are calculated using Eq (17) and the first group of low-frequency modes. In the figure, capsid proteins are shown in ribbon representation. In order to highlight pentamers and their locations in the figure, the first residues of the capsid proteins in pentamers are rendered as red spheres. They would have been colored in dark blue otherwise since their MSFs are smaller than 7.1 Å^2^. The figures show that the MSFs of hexamers gradually increase as they move away from pentamers, as indicated by the change in color.

Fig 10(D) quantifies the trend that MSF increases as hexamers move away from pentamers. In the figure, each pixel represents *C*^*α*^ atoms of the hexamers with the given MSF (the ordinate axis) and the distance to the closest pentamer (the abscissa axis). Each pixel’s gray level represents the number of *C*^*α*^ atoms, ranging from zero (in white) to as many as 528 (in black). The plot shows a strong linear correlation, indicating that the MSF of a hexamer increases as its distance to the closest pentamer increases.

Our result indicates that while the structural role of pentamers is forming both hemispherical ends of the capsule-shaped HIV-1 capsid shell [38, 39], the dynamic role of pentamers is to stabilize both ends of the capsid by suppressing their fluctuations. Our result is in agreement with the idea that capsid disassembly should start when the pentamers become destabilized [40]. The narrow end of the cone especially, where pentamers are more concentrated, was thought to be the place where destabilization is triggered and disassembly begins [39]. The recent work by Rankovic et al. [41] using atomic force microscopy (AFM) confirmed this.

#### The transition from global motions to localized motions

Fig 9(B) shows the MSFs of capsid proteins determined using the first two mode groups: the first 700 modes (blue) and those up to 3,000th modes (red). In the figure, the blue line shows that the first group of modes contribute about evenly to all the residues, while the second group of modes as represented by the red line contribute mostly to a localized region around the N-terminal loop (residues 85-93). Therefore, there is a distinct transition from a global motion to a more localized motion as we move from the first group of modes to the second.

**Fig 11:**
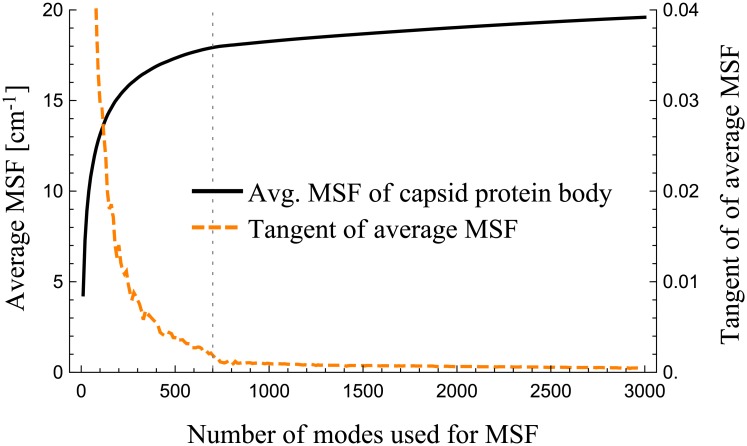
The fluctuation dynamics of the capsid protein body and how it varies over the modes.

Fig 11 further elucidates this transition. In the figure, the black line shows how the cumulative squared fluctuations of the body of capsid proteins (residues 20–74 and 104–220) change over the modes. The orange dashed line shows the slope, or the rate of change, of the black line. The plot shows that the magnitude of motions of the body of the capsid proteins nearly vanishes at the end of the first mode group, at a frequency around 1.3 cm^-1^.

#### The swing motions of the NTD loops in the second mode group

**Fig 12:**
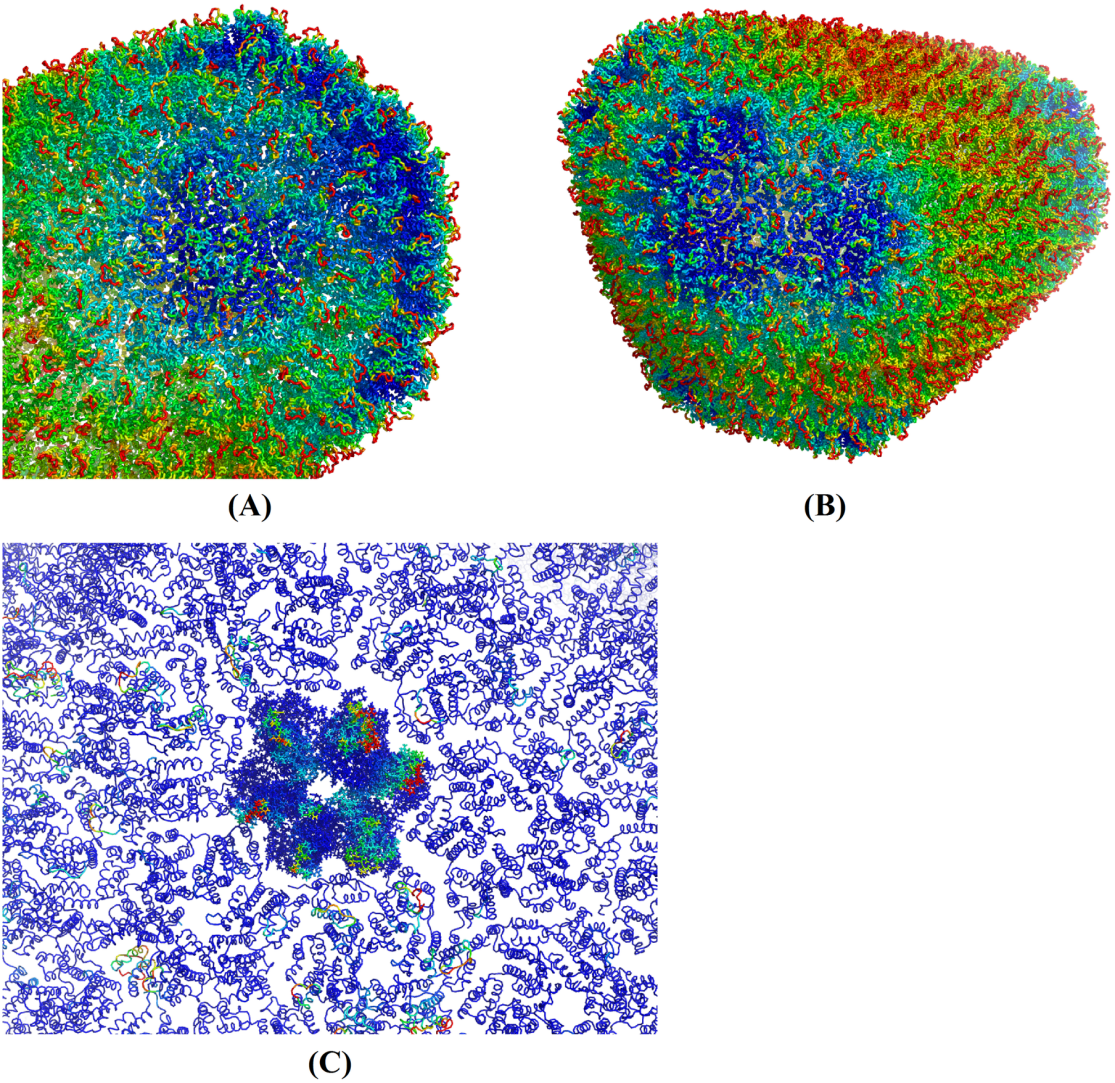
Fluctuation dynamics of HIV-1 capsid as determined using the first 3,000 modes. (A) and (B) show the HIV-1 capsid structure in different orientation, color-coded by MSFs determined using the first 3,000 modes. Dark blue represents MSFs that are smaller than 6.0 Å^2^, while red represents MSFs larger than 42.9 Å^2^. (C) Part of the capsid that is color-coded by the squared fluctuations of one mode only (the 3,000^th^ mode), which is different from (A) and (B). It highlights one hexamer by showing it in an all-atom representation. A movie file that captures the loop swing motion in this mode is available at S1 Video.

Fig 9(B) shows also that the second group of modes (red region, 1.3–2.3 cm^-1^) is mostly about the fluctuation dynamics of the N-terminal loop. Fig 12(A) and 12(B) display the HIV-1 capsid color-coded by MSFs, determined using the first 3,000 modes. In the figure, dark blue and red represent MSFs smaller than 6.0 Å^2^ and larger than 42.9 Å^2^, respectively. The figure shows that MSFs of almost all the loops on the surface of HIV-1 capsid are high (red) while MSFs of capsid protein bodies underneath the loops remain similar to those in Fig 10. This implies again that modes in 1.3–2.3 cm^-1^ region (the second group of modes) are about the swing motions of the loops in NTD. Fig 12(C) shows the squared fluctuations in one of the modes in the second group (the 3,000^*th*^ mode), with one hexamer highlighted in its all-atom line representation. A visual inspection of this mode reveals again the apparent swing motions of the NTD loops. The motion of this mode is captured in a movie file and is available at S1 Video.

#### The dynamic role of hexamer pores in nucleotide transport

It was proposed by Jacques et al. [42] that HIV-1 uses the hexamer pores to import nucleotides needed for DNA synthesis. The pore of each hexamer is formed by six N-terminal *β*-hairpins. In the open state, the pore is about 25 Å deep and has a volume of 3,240 Å^3^ [42]. At the bottom of the pore is an arginine ring of 6 formed by residues 18. The arginine ring was shown to play a significant role in nucleotide transport and was thought to recruit dNTPs (deoxynucleoside triphosphates) and then release them into the interior of the capsid, to be used for DNA synthesis [42].

Pentamers on the HIV-1 capsid, on the other hand, must have a lesser role in the nucleotide transport process. They count for less than five percent of the total number of capsomeres. Furthermore, they have a significantly lower magnitude of motions than the hexamers (Fig 10). The idea that hexamers contribute most of the transport was supported also in studies of bacterial microcompartments [43, 44].

The all-atom dynamics of the HIV-1 capsid produced by BOSE offer a great opportunity to study the dynamics of the pores. The normal modes allow us to examine the fluctuations of the radii of the arginine rings and to ask a number of interesting questions regarding the transport. Do all hexamers participate in the nucleotide transport? If only a subset of hexamers do, where are they located in the surface of the capsid? From Fig 10(D) we see the magnitude of fluctuations of the hexamers is greater when they are further away from pentamers. Does this mean hexamers participating in the transport also are away from the pentamers?

To examine the radii of arginine rings (there are 216 hexamers and thus the same number of arginine rings) and their fluctuation dynamics, we perform the following computations. Let **a**_*i*_ (1 ≤ *i* ≤ 6) be the coordinates of the *C*^*α*^ atoms of any given arginine ring of 6 whose mass center is already shifted to the origin. The radius *r* of the ring can be calculated as:

$$\begin{array}{c} r=\min_{1\leq i\leq 6}(∥a_{i}∥), \end{array}$$ (18)

i.e., the radius is the same as the distance between the closest arginine and the center. Note that side chains are not considered for simplicity, as the local dynamics and rearrangements of the side chains are a different matter from the effect of the global dynamics that will be focused on in the following.

Next, we consider how the low frequency normal modes of the whole capsid computed earlier by BOSE affect the radii of arginine rings that are distributed over the capsid. Specifically, we compute for each arginine ring, what combination of modes and their resulting displacement are able to bring the largest increment to its radius. This is done in two steps. We first compute numerically the gradient of the ring radius relative to the modes **v**, or $\nabla_{v}^{r}$. Then for any displacement **d** = ∑_*i*_*c*_*i*_**v**_*i*_ where *c*_*i*_ is the component of the displacement along the *i*^*th*^ mode **v**_*i*_, we compute:

$$\begin{array}{c} \text{arg}\underset{c}{\text{max}}\;c\cdot \nabla_{v}^{r}\;, \end{array}$$ (19)

subject to

$$\begin{array}{c} \sum_{i}c_{i}^{2}\lambda_{i}=const\;, \end{array}$$ (20)

where **c** is a vector composed of *c*_*i*_’s. The above constraint ensures that all displacements should cost an equal amount of energy. Specifically, *const* is chosen such that the structure deviation along the slowest mode is 3 Å. The first 3,000 low frequency modes (which belong to the first two mode groups aforementioned) are used in the computation in Eq (19) since they are of high quality. Using all 34,200 BOSE modes gives a similar result.

**Fig 13:**
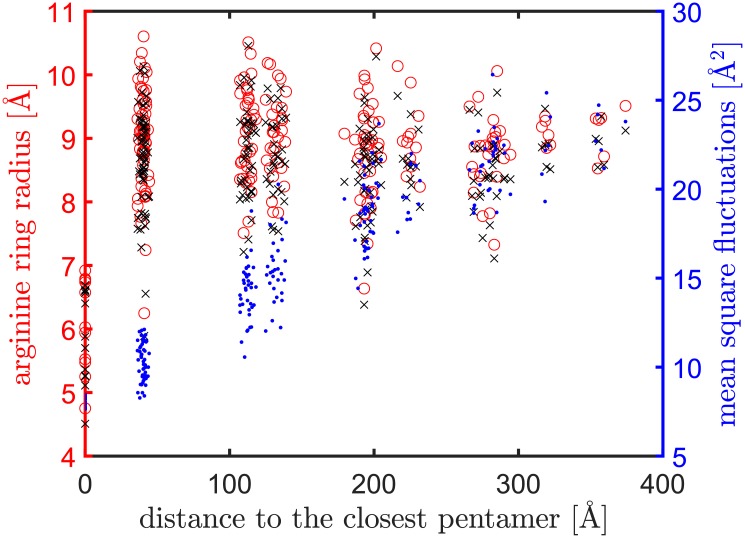
The fluctuation dynamics of the arginine rings determined using the first 3,000 modes. The radii of the arginine rings at the initial structure are marked by black crosses. Red open circles mark what the radii become after thermal fluctuations are considered. Along the abscissa axis, the data points (representing the arginine rings) are separated into several distinct groups based on their distances to the closest pentamer. Average MSFs of the arginine rings are marked by blue dots.

Fig 13 shows the results. The radii of the arginine rings at the initial structure (pdb-id: 3J3Q) [13] are marked by black crosses. The red open circles mark the new radii of the rings after taking a local displacement (a certain combination of normal modes) that gives the largest increment in the radius. The blue dots represent the average MSFs of the arginine rings, which shows that the magnitude of thermal fluctuations of the arginine rings increases proportionally as they move away from pentamers, in agreement with what was seen in Fig 10(D). Along the abscissa axis of Fig 13, the arginine rings fall into several distinct groups based on their distances to the closest pentamer. The first group is of the pentamers themselves (distance = 0); the second group is of hexamers right next to the pentamers (distance ≈ 40 Å); the third group is of those hexamers whose distances are between 100 to 150 Å away from the closest pentamer: these are hexamers that are adjacent to the hexamers in the second group; lastly, the remaining data points represent hexamers that are further away from the pentamers.

Fig 13 shows within each group the radius variations caused by thermal fluctuations (from black crosses to red open circles). Surprisingly, though mean square fluctuations (MSFs) of arginine rings increase quickly as they move away from pentamers (blue dots in Fig 13), the radii of arginine rings fluctuate more within a group of hexamers that are *closer* to pentamers. The largest radius reachable within each group also is greater when a group is closer to pentamers. Since the reachable radii of the arginine rings are presumably proportional to the functional activity of the rings, we predict, based on these evidences, that not all hexamers on the HIV-1 capsid participate equally in the nucleotide transport: more nucleotide transport should take place at hexamers *nearer* to pentamers. A possible explanation is that the low MSFs of pentamers offer the needed dynamic stability to the nearby hexamers for nucleotide transport, while hexamers far from pentamers have too large fluctuations to be effective in the transport. In other words, hexamers far from pentamers may have large fluctuations as a whole but rather small intramolecular motions to transport nucleotides through their arginine rings. Additionally, we notice that among the arginine rings that have the largest radii, say greater than 10 Å (there are 13 such open circles in Fig 13), most of them (10 out of 13) are located at the larger hemispherical end of the capsid. Thus it is possible that nucleotide transport happens dominantly at the larger hemispherical end, which is reasonable considering that the larger end has more leeway for DNA synthesis to take place. Lastly, it is evident from Fig 13 that pentamers definitely do not participate in nucleotide transport since the radii of their arginine rings are significantly smaller. Their role thus must be about stabilizing the whole capsid, as aforementioned.

## Discussion

In this work, we have demonstrated that the BOSE model, which is based on the principle of resonance discovered in our previous work [1], is suited for efficiently computing the all-atom normal modes of extremely large assemblies through a novel projection-based approach that preserves the accuracy in dynamics. The principle of resonance is derived from our observation that the vibrations of a whole capsid at any given frequency *ω* is contributed mostly by vibrations of component capsomeres at around the same frequency [1]. This resonance principle is the foundation of the BOSE model and makes it possible to *efficiently* and *accurately* compute the vibrations of a whole capsid at any given frequency by projecting the motions of component capsomeres into a narrow subspace. We have conducted the assessments of the quality of the BOSE modes by comparing them with benchmark ANM modes and sbNMA modes obtained directly from the original Hessian matrices. The assessments further underline the importance of a proper modeling of the elasticity of each capsomere, as is done in BOSE using a selected subset of normal modes in the right range of frequency, and show that a model with a proper modeling of capsomeres’ elasticity produces significantly better normal modes than models without.

HIV-1 capsid is an extremely large system that is composed of nearly 5 million atoms. There were several computational studies of its dynamics using coarse-grained MD simulation [29], PCA [28, 30], and RTB [11]. In the PCA approach, even though conformations were obtained from all-atom MD simulations, coarse-grained representations of the system were used in determining the global motions of the whole structure. In the RTB approach, coarse-grained ANM was used as the base model. Here, for the first time to our knowledge, we have determined the all-atom normal mode dynamics of an entire HIV-1 capsid including hydrogens by employing the proposed BOSE model.

Our results reveal some interesting insights into the dynamics of this large capsid. First, we observe that there is a clear distinction between two groups of modes at the low frequency end of the vibrational spectrum. A close examination shows that the first group of modes are mostly about global fluctuations while the second group of modes are mostly about local swing motions of the N-terminal loop of the capsid proteins. Second, we identify the dynamic role of the twelve pentamers on the capsid. While the structural role of the pentamers was thought to form the hemispherical ends of the capsule-shaped HIV-1 capsid [38, 39], analysis of the first group of modes and the associated global fluctuations reveal what the dynamic role of the pentamers is: pentamers serve to stabilize both ends of the capsid dynamically by suppressing the fluctuation dynamics of the capsid at around their locations. This is consistent with the results obtained from MD simulation [28]. Our result thus indirectly supports the idea that capsid disassembly or uncoating may start when the pentamers becomes unstabilized [40], and the narrow end of the cone may be the place where destabilization is triggered and disassembly begins [39]. A recent work by Rankovic et al. [41] using atomic force microscopy (AFM) further confirmed that HIV-1 capsid underwent rupture near the narrow end of the capsid. Lastly, our results on the dynamics of hexamer pores suggest that nucleotide transport should take place mostly at hexamers near pentamers, especially at the larger hemispherical end.

### Even larger capsids and assemblies

Though the HIV-1 capsid studied in this work is one of the largest structures determined so far [12], it is expected that atomic structures of even larger assemblies will come into light in the near future, such as structures of some of the bacterial microcompartments [43], which are known to be made up of thousands of protein chains [43, 44]. Faustovirus (pdb-id: 5J7V) [45] is another example. It has an astounding number, 8,280 to be precise, of chains. How can we ready ourselves for the dynamics studies of these giant assemblies? A possible way to manage their immense size is to employ a hierarchical modeling of the whole structure. Specifically, a whole capsid may be first divided into fragments, with each fragment piece composed of a manageable number of capsomeres. Once this hierarchical structure is set up, one may apply the principle of resonance iteratively by obtaining first the dynamics of fragments from those of capsomeres and then the dynamics of the whole capsid from those of fragments. Such studies may help also pave the way for future simulations of organelles and even of cells.

### Applying BOSE to non-capsid assemblies

In this work, BOSE is applied solely to homomeric capsids. It is foreseeable that it can be easily extended to heteromeric capsids with minor adjustment and possibly, even to non-capsid assemblies. When a biomolecular system is composed of different proteins or even nucleic acids, different approaches may need to be combined. For example, when studying the ribosome that is made up of ribosomal RNAs and several dozens of distinct proteins, elastic units can be selected by considering the sizes of RNAs and proteins and their structural shapes, and the number of modes may be selected according to the size of each unit. We plan to extend BOSE to study such systems in future work.

### Pore regulation and cooperativity in capsids

HIV-1 capsid uses the central pores of its hexamers to import nucleotides and to fuel encapsidated DNA synthesis [42]. The pores of the hexamers were thought to undergo an iris-like opening and closing motion [42]. Are the iris-like motions of the pores totally uncorrelated or fully synchronized somehow, or somewhere in between? Currently little is known and it is certainly worth investigating. Note that HIV-1 capsid in this regard resembles closely bacterial microcompartments (MCP), which also are enveloped by structural shells that are fully proteinaceous. The capsids of MCP serve as a diffusion barrier that isolates toxic reaction intermediates from the cytoplasm while allowing substrates, co-factors, and products to pass through [43, 44]. The MCP capsids are composed of up to a few thousand shell proteins, most of which form hexamers or pseudo-hexamers (trimers) with central pores that are important functionally and are regulated dynamically. The dynamic regulation of MCP pores again is not well understood and normal mode analysis of these systems may provide the needed insights. It should be noted that at present the atomic structures of most MCP capsids are yet unknown except for a few, including the recent determined shell structure from *Haliangium ochraceum* [14].

## Supporting information

S1 Video Video of the NTD loop motion as revealed in a normal mode of HIV-1 capsid. This video captures the NTD loop motion in Fig 12(C). In the video, one hexamer is highlighted and shown in an all-atom representation while the surrounding proteins are in ribbon representation. The structure is color-coded by MSFs determined using only the 3,000th mode.
(MPG)

S1 Appendix BOSE normal mode computation when the mass matrix is present. (PDF)

## References

[1] Na H, Song G. Fast Normal Mode Computations of Capsid Dynamics Inspired by Resonance. Phys Biol. 2018; 10.1088/1478-3975/aab813 29557348

[2] Frank J. Advances in the field of single-particle cryo-electron microscopy over the last decade. Nat Protoc. 2017;12:209–212. 10.1038/nprot.2017.004 28055037 PMC5479931

[3] Go N, Noguti T, Nishikawa T. Dynamics of a small globular protein in terms of low-frequency vibrational modes. Proc Natl Acad Sci USA. 1983;80(12):3696–3700. 10.1073/pnas.80.12.3696 6574507 PMC394117

[4] Brooks B, Karplus M. Harmonic dynamics of proteins: normal modes and fluctuations in bovine pancreatic trypsin inhibitor. Proc Natl Acad Sci USA. 1983;80(21):6571–6575. 10.1073/pnas.80.21.6571 6579545 PMC391211

[5] Levitt M, Sander C, Stern PS. The Normal Modes of a protein: Native bovine Pancreatic Trypsin inhibitor. Int J Quant Chem. 1983;10:181–199.

[6] Lehoucq RB, Sorensen DC, Yang C. ARPACK Users Guide: Solution of Large-Scale Eigenvalue Problems with Implicitly Restarted Arnoldi Methods. SIAM, Philadelphia; 1998.

[7] Dykeman EC, Sankey OF. Low Frequency Mechanical Modes of Viral Capsids: An Atomistic Approach. Phys Rev Lett. 2008;100:028101 10.1103/PhysRevLett.100.028101 18232930

[8] Dykeman EC, Sankey OF. Atomistic modeling of the low-frequency mechanical modes and Raman spectra of icosahedral virus capsids. Phys Rev E. 2010;81:021918 10.1103/PhysRevE.81.021918 20365606

[9] Tama F, Gadea FX, Marques O, Sanejouand YH. Building-block approach for determining low-frequency normal modes of macromolecules. Proteins. 2000;41:1–7. 10.1002/1097-0134(20001001)41:1%3C1::AID-PROT10%3E3.0.CO;2-P 10944387

[10] Li G, Cui Q. A coarse-grained normal mode approach for macromolecules: an efficient implementation and application to Ca(2+)-ATPase. Biophys J. 2002;83:2457–2474. 10.1016/S0006-3495(02)75257-0 12414680 PMC1302332

[11] Bergman S, Lezon TR. Modeling Global Changes Induced by Local Perturbations to the HIV-1 Capsid. J Mol Graph Model. 2017;71:218–226. 10.1016/j.jmgm.2016.12.003 27951510

[12] Berman HM, Westbrook J, Feng Z, Gilliland G, Bhat TN, Weissig H, et al The Protein Data Bank. Nucleic Acids Res. 2000;28(1):235–42. 10.1093/nar/28.1.235 10592235 PMC102472

[13] Zhao G, Perilla JR, Yufenyuy EL, Meng X, Chen B, Ning J, et al Mature HIV-1 capsid structure by cryo-electron microscopy and all-atom molecular dynamics. Nature. 2013;497:643–646. 10.1038/nature12162 23719463 PMC3729984

[14] Sutter M, Greber B, Aussignargues C, Kerfeld CA. Assembly principles and structure of a 6.5-MDa bacterial microcompartment shell. Science. 2017;356(6344):1293–1297. 10.1126/science.aan3289 28642439 PMC5873307

[15] Atilgan AR, Durell SR, Jernigan RL, Demirel MC, Keskin O, Bahar I. Anisotropy of Fluctuation Dynamics of Proteins with an Elastic Network Model. Biophys J. 2001;80(1):505–515. 10.1016/S0006-3495(01)76033-X 11159421 PMC1301252

[16] Na H, Song G. The Effective Degeneracy of Protein Normal Modes. Phys Biol. 2016;13(3):036002 10.1088/1478-3975/13/3/036002 27171157

[17] Yang L, Song G, Jernigan RL. How Well Can We Understand Large-Scale Protein Motions Using Normal Modes of Elastic Network Models? Biophys J. 2007;93:920–929. 10.1529/biophysj.106.095927 17483178 PMC1913142

[18] Humphrey W, Dalke A, Schulten K. VMD—Visual Molecular Dynamics. J Mol Graphics. 1996;14:33–38. 10.1016/0263-7855(96)00018-5 8744570

[19] Phillips JC, Braun R, Wang W, Gumbart J, Tajkhorshid E, Villa E, et al Scalable molecular dynamics with NAMD. Journal of Computational Chemistry. 2005;26:1781–1802. 10.1002/jcc.20289 16222654 PMC2486339

[20] Na H, Song G. Bridging between normal mode analysis and elastic network models. Proteins. 2014;82:2157–2168. 10.1002/prot.24571 24692201

[21] MacKerell AD, Bashford D, Bellott, Dunbrack RL, Evanseck JD, Field MJ, et al All-Atom Empirical Potential for Molecular Modeling and Dynamics Studies of Proteins. J Phys Chem B. 1998;102(18):3586–3616. 10.1021/jp973084f 24889800

[22] Wang J, Cieplak P, Kollman PA. How well does a restrained electrostatic potential (RESP) model perform in calculating conformational energies of organic and biological molecules? J Comput Chem. 2000;21(12):1049–1074. 10.1002/1096-987X(200009)21:12%3C1049::AID-JCC3%3E3.0.CO;2-F

[23] Na H, Song G. A natural unification of GNM and ANM and the role of inter-residue forces. Phys Biol. 2014;11(3):036002 10.1088/1478-3975/11/3/036002 24732806

[24] Na H, Jernigan RL, Song G. Bridging between NMA and Elastic Network Models: Preserving All-atom Accuracy in Coarse-grained Models. PLoS Comput Biol. 2015;11(10):e1004542 10.1371/journal.pcbi.1004542 26473491 PMC4608564

[25] Na H, Song G. Predicting the Functional Motions of p97 Using Symmetric Normal Modes. Proteins. 2016;84:1823–1835. 10.1002/prot.25164 27653958

[26] Na H, Song G, Ben-Avraham D. Universality of Vibrational Spectra of Globular Proteins. Phys Biol. 2016;13(1):016008 10.1088/1478-3975/13/1/016008 26907186

[27] Eyal E, Yang LW, Bahar I. Anisotropic network model: systematic evaluation and a new web interface. Bioinformatics. 2006;22(21):2619–2627. 10.1093/bioinformatics/btl448 16928735

[28] Perilla JR, Schulten K. Physical properties of the HIV-1 capsid from all-atom molecular dynamics simulations. Nat Commun. 2017;8:15959 10.1038/ncomms15959 28722007 PMC5524983

[29] Grime JMA, Dama JF, Ganser-Pornillos BK, Woodward CL, Jensen GJ, Yeager M, et al Coarse-grained simulation reveals key features of HIV-1 capsid self-assembly. Nat Commun. 2016;7:11568 10.1038/ncomms11568 27174390 PMC4869257

[30] Noel JK, Levi M, Raghunathan M, Lammert H, Hayes RL, Onuchic JN, et al SMOG 2: A Versatile Software Package for Generating Structure-Based Models. PLoS Comput Biol. 2016;12(3):e1004794 10.1371/journal.pcbi.1004794 26963394 PMC4786265

[31] Lane SW, Dennis CA, Lane CL, Trinh CH, Rizkallah PJ, Stockley PG, et al Construction and Crystal Structure of Recombinant STNV Capsids. Journal of Molecular Biology. 2011;413(1):41–50. 10.1016/j.jmb.2011.07.062 21839089

[32] Gulati A, Murthy A, Abraham A, Mohan K, Natraj U, Savithri HS, et al Structural studies on chimeric Sesbania mosaic virus coat protein: Revisiting SeMV assembly. Virology. 2016;489:34–43. 10.1016/j.virol.2015.11.029 26704627

[33] Chen NC, Yoshimura M, Guan HH, Wang TY, Misumi Y, Lin CC, et al Crystal Structures of a Piscine Betanodavirus: Mechanisms of Capsid Assembly and Viral Infection. PLoS Pathog. 2015;11(10):e1005203 10.1371/journal.ppat.1005203 26491970 PMC4619592

[34] Sasaki E, Böhringer D, van de Waterbeemd M, M L, Zschoche R, Heck AJ, et al Structure and assembly of scalable porous protein cages. Nat Commun. 2017;8:14663 10.1038/ncomms14663 28281548 PMC5354205

[35] Song G. Symmetry in normal modes and its strong dependence on symmetry in structure. J Mol Graphics Modell. 2017;75:32–41. 10.1016/j.jmgm.2017.04.002 28501531

[36] van Vlijmen HW, Karplus M. Normal mode analysis of large systems with icosahedral symmetry: application to (Dialanine)60 in full and reduced basis set. J Chem Phys. 2001;115(2):691–698. 10.1063/1.1370956

[37] van Vlijmen HW, Karplus M. Normal mode calculations of icosahedral viruses with full dihedral flexibility by use of molecular symmetry. J Mol Biol. 2005;350(3):528–42. 10.1016/j.jmb.2005.03.028 15922356

[38] Chen B. HIV Capsid Assembly, Mechanism, and Structure. Biochemistry. 2016;55:2539–2552. 10.1021/acs.biochem.6b00159 27074418

[39] Pornillos O, Ganser-Pornillos BK, Yeager M. Atomic level modeling of the HIV capsid. Nature. 2011;469:424–427. 10.1038/nature09640 21248851 PMC3075868

[40] Zandi R, Reguera D. Mechanical properties of viral capsids. Phys Rev E. 2005;72:021917 10.1103/PhysRevE.72.021917 16196614

[41] Rankovic S, Varadarajan J, Ramalho R, Aiken C, Rousso I. Reverse Transcription Mechanically Initiates HIV-1 Capsid Disassembly. J Virol. 2017;91(12):e00289–17. 10.1128/JVI.00289-17 28381579 PMC5446659

[42] Jacques DA, McEwan WA, Hilditch L, Price AJ, Towers GJ, James LC. HIV-1 uses dynamic capsid pores to import nucleotides and fuel encapsidated DNA synthesis. Nature. 2016;536:349–353. 10.1038/nature19098 27509857 PMC4998957

[43] Yeates TO, Thompson MC, Bobik TA. The protein shells of bacterial microcompartment organelles. Curr Opin Struct Biol. 2011;21(2):223–231. 10.1016/j.sbi.2011.01.006 21315581 PMC3070793

[44] Chowdhury C, Chun S, Pang A, Sawaya MR, Sinha S, Yeates TO, et al Selective molecular transport through the protein shell of a bacterial microcompartment organelle. Proc Natl Acad Sci USA. 2015;112(10):2990–2995. 10.1073/pnas.1423672112 25713376 PMC4364225

[45] Klose T, Reteno DG, Benamar S, Hollerbach A, Colson P, La Scola B, et al Structure of faustovirus, a large dsDNA virus. Proc Natl Acad Sci USA. 2016;113(22):6206–6211. 10.1073/pnas.1523999113 27185929 PMC4896704

