# Supplementary material for: All-atom normal mode dynamics of HIV-1 capsid
Source: PLoS Comput Biol. 2018 Sep 18;14(9):e1006456. doi: 10.1371/journal.pcbi.1006456 (PMC6161923; doi:10.1371/journal.pcbi.1006456)
Supplement: S1 Appendix — (PDF) [file pcbi.1006456.s002.pdf]

All-atom Normal Mode Dynamics of HIV-1 Capsid

Hyuntae Na[1*], Guang Song[2,3]

1 Department of Computer Science, Penn State Harrisburg, Middletown, PA 17057, USA
2 Department of Computer Science, Iowa State University, Ames, Iowa 50011, USA
3 Program of Bioinformatics and Computational Biology, Iowa State University, Ames, Iowa 50011, USA

*** Corresponding author**
hzn17@psu.edu (HN)

S1 Appendix.

BOSE normal mode computation when the mass matrix is present.

First, denote by m the total number of blocks in a system, and by n the number of atoms in each block. Let $\mathbf{H}^{(i)}$ be the $3n \times 3n$ Hessian matrix of the i-th block in isolation, and $\mathbf{M}^{(i)}$ be its $n \times n$ diagonal mass matrix, where $i = 1, ..., m$. Note that the superscript with parenthesis "$^{(i)}$" represents the i-th block. The j-th mode $\mathbf{v}_j^{(i)}$ of the i-th block is determined by solving the following generalized eigenvalue problem:

$$\mathbf{H}^{(i)}\,\mathbf{v}_j^{(i)} = \lambda_j^{(i)}\left(\mathbf{M}^{(i)} \otimes \mathbf{I}_3\right)\mathbf{v}_j^{(i)} ,$$

where $\otimes$ is the operator of the Kronecker product, $\mathbf{I}_k$ is the $k \times k$ identity matrix, and $\lambda_j^{(i)}$ is the corresponding eigenvalue of the j-th eigenvector $\mathbf{v}_j^{(i)}$, where $\lambda_j^{(i)} \le \lambda_k^{(i)}$ for $\forall j < k$. The elasticity matrix $\mathbf{J}^{(i)}$ of the i-th block is defined, as follows, in a form of $3n \times l$ matrix:

$$\mathbf{J}^{(i)} = \left(\frac{\mathbf{v}_1^{(i)}}{\|\mathbf{v}_1^{(i)}\|}, \frac{\mathbf{v}_2^{(i)}}{\|\mathbf{v}_2^{(i)}\|}, \cdots, \frac{\mathbf{v}_l^{(i)}}{\|\mathbf{v}_l^{(i)}\|}\right) ,$$

where l is the number of modes to use for each block, and $\|\mathbf{p}\|$ is the second norm of a vector $\mathbf{p}$.

The elasticity matrix $\mathbf{J}$ of the whole system is defined by combining $\mathbf{J}^{(1)}, ..., \mathbf{J}^{(m)}$, as follows, in a form of $3nm \times lm$ block diagonal matrix:

$$\mathbf{J} = \begin{pmatrix} \mathbf{J}^{(1)} & \mathbf{0} & \cdots & \mathbf{0} \\ \mathbf{0} & \mathbf{J}^{(2)} & \cdots & \mathbf{0} \\ \vdots & \vdots & \ddots & \vdots \\ \mathbf{0} & \mathbf{0} & \cdots & \mathbf{J}^{(m)} \end{pmatrix} .$$

In the above, each column vector of $\mathbf{J}$ is a $3nm \times 1$ unit vector. Let $\mathbf{H}$ be a $3nm \times 3nm$ (sparse) Hessian matrix of the system in the Cartesian coordinates. The reduced Hessian matrix $\tilde{\mathbf{H}}$ is defined, using $\mathbf{J}$, as follows:

$$\tilde{\mathbf{H}} = \mathbf{J}^\top \mathbf{H} \mathbf{J} ,$$

where $\top$ stands for transpose.

Let $\tilde{\mathbf{v}}_j$ and λ_j be the j-th eigenvector and its corresponding eigenvalue, respectively, obtained by solving the following generalized eigenvalue problem:

$$\tilde{\mathbf{H}}\tilde{\mathbf{v}}_i = \lambda_i \tilde{\mathbf{M}}\tilde{\mathbf{v}}_i \ ,$$

where $\tilde{\mathbf{M}}$ is defined, using the $nm \times nm$ diagonal mass matrix $\mathbf{M}$ of the whole system, as follows:

$$\tilde{\mathbf{M}} = \mathbf{J}^\top (\mathbf{M} \otimes \mathbf{I}_3) \mathbf{J} \ .$$

In actual implementation, $\tilde{\mathbf{H}}$ and $\tilde{\mathbf{M}}$ should be computed block by block as in Eq (8) to save memory and computational time.

Now the i-th mode $\mathbf{v}_i$ of the whole system in the Cartesian coordinates can be obtained as follows:

$$\mathbf{v}_i = \mathbf{J}\tilde{\mathbf{v}}_i \ .$$
